# Effects of Multispecies Probiotic on Intestinal Microbiota and Mucosal Barrier Function of Neonatal Calves Infected With *E. coli* K99

**DOI:** 10.3389/fmicb.2021.813245

**Published:** 2022-01-26

**Authors:** Yanyan Wu, Cunxi Nie, Ruiqing Luo, Fenghua Qi, Xue Bai, Hongli Chen, Junli Niu, Chen Chen, Wenju Zhang

**Affiliations:** ^1^College of Animal Science and Technology, Shihezi University, Shihezi, China; ^2^Xinjiang Tianshan Junken Animal Husbandry Co., Ltd., Shihezi, China

**Keywords:** multispecies probiotic, immune function, microbiota function, neonatal calves, *E. coli* K99

## Abstract

Altered gut microbiota are implicated in inflammatory neonatal calf diarrhea caused by *E. coli* K99. Beneficial probiotics are used to modulate gut microbiota. However, factors that mediate host-microbe interactions remain unclear. We evaluated the effects of a combination of multispecies probiotics (MSP) on growth, intestinal epithelial development, intestinal immune function and microbiota of neonatal calves infected with *E. coli* K99. Twelve newborn calves were randomly assigned as follows: C (control, without MSP); D (*E. coli* O78:K99 + gentamycin); and P (*E. coli* O78:K99 + supplemental MSP). All groups were studied for 21 d. MSP supplementation significantly (i) changed fungal Chao1 and Shannon indices of the intestine compared with group D; (ii) reduced the relative abundance of *Bacteroides* and *Actinobacteria*, while increasing *Bifidobacteria*, *Ascomycetes*, and *Saccharomyce*s, compared with groups C and D; (iii) improved duodenal and jejunal mucosal SIgA and total Short Chain Fatty Acids (SCFA) concentrations compared with group D; (iv) increased relative *ZO-1* and *occludin* mRNA expression in jejunal mucosa compared with group D; and (v) enhanced intestinal energy metabolism and defense mechanisms of calves by reducing HSP90 expression in *E. coli* K99, thereby alleviating the inflammatory response and promoting recovery of mucosal function. Our research may provide direct theoretical support for future applications of MSP in ruminant production.

## Introduction

Newborn calf diarrhea (NCD) can cause huge economic losses due to high morbidity and mortality (Walker et al., 1998; Cho and Yoon, 2014; Brunauer et al., 2021). The risk factors associated with diarrhea include the environmental conditions, nutritional levels, and immune status of calves (Klein-Jöbstl et al., 2014; Al Mawly et al., 2015). The most common enteric pathogens include *Escherichia coli*, *Salmonella*, *Cryptosporidium* and *Rotavirus* (Moon et al., 1978; Gulliksen et al., 2009; da Silva Medeiros et al., 2015). Enterotoxigenic *Escherichia coli* (ETEC), which constitute the most common cause of neonatal calf diarrhea globally, have been extensively studied over the past 40 years (Bywater and Logan, 1974; González Pasayo et al., 2019). Neonatal calves are most susceptible *E. coli* K99 ETEC infections during the first 4 days of life (Krogh, 1983; Acres, 1985).

The *E. coli* K99 antigen binds to the small intestinal mucosa and gradually decreases from the first 12 h of adhesion. This adhesion ability is related to age (Runnels et al., 1980). However, in ETEC, this ability increases after the 3rd week of age (Bulgin et al., 1982; Izzo et al., 2011). Antibiotics have been used to treat *E. coli* that cause diarrhea (Sunderland et al., 2003), where these antibiotics not only affect the target pathogens, but also beneficial microorganisms in the intestine, resulting in long-term changes in intestinal microbiota being associated with the disease. Application of antibiotics also exerts many other side effects, such as intestinal barrier dysfunction and the emergence of carcinogenic and drug-resistant bacteria, which greatly affect the usefulness of antibiotics (Raheem et al., 2021). Probiotics, which are considered as sustainable alternatives to antibiotics, can be used to prevent and treat diarrhea in humans and animals (Collinson et al., 2020; Dahlgren et al., 2021; Hrala et al., 2021). Many previous studies have demonstrated that supplementing calves with probiotics early in life effectively prevents diarrhea (Malmuthuge and Guan, 2017; Wu et al., 2021a). *Lactobacillus acidophilus* (Lépine et al., 2018), *Bacillus subtilis* (Sambanthamoorthy et al., 2014; Shen et al., 2017; Memon et al., 2021) and *Saccharomyces cerevisiae* (Chiaro et al., 2017; Bitla et al., 2021) has the ability to resist pathogen adhesion and enhance the intestinal barrier function (Sanchez et al., 2010; Younis et al., 2017).

In this study, we evaluated whether newborn calves were infected with *E. coli* K99, and whether supplementation with MSP enhanced the integrity of the intestinal barrier and local and systemic immune responses of calves, by regulating intestinal microbiota and ameliorating intestinal dysfunction caused by inflammation. In addition, we investigated the recovery of damaged intestinal function and explored the interaction between the diversity of microbiota and the repair of intestinal mucosa.

## Materials and Methods

### Preparation of Multispecies Probiotics Complex Preparations

The MSP (*L. acidophilus* 3 × 10^9^ CFU/1 g, *B. subtilis* 3 × 10^9^ CFU/1 g, and *S. cerevisiae* 1 × 10^9^ CFU/1 g) was prepared by the Biological Feed Laboratory of the College of Animal Science and Technology, Shihezi University, and the MSP preparation method is described in the previous study (Wu et al., 2021b).

### Experimental Design

Thirty-six male Holstein calves (body weight 40.1 ± 0.6 kg; age 5 ± 2) were used for this study. Three groups, each containing 12 calves that were randomly assigned using a random number generator (Microsoft Corp., Redmond, WA), were formed as follows: (i) the control group (C) fed a basal diet and not challenged with *E. coli* K99; (ii) the diarrhea group (D) also fed a basal diet and orally challenged with *E. coli* K99 (30 mL; 1 × 10^9^ CFU/mL) and antibiotic support therapy (intramuscular gentamicin 20 mL/days, injection lasted for 2 days); and (iii) the MSP group (P): fed MSP every day from the next day (7.0 × 10^9^CFU/g; 2 g/calf) after orally challenged with *E. coli* K99 (30 mL; 1.0 × 10^9^CFU/mL). This is because the best effect was obtained with 2 g MSP per day according to a previous study of ours (Wu et al., 2021a), MSP which is in the form of a powder, was administered via milk. The basal diet (Supplementary Table 1) was free of antibiotics. The experimental treatments lasted 21 d, during which all the animals had free access to fresh water and starter concentrate. The starter concentrate, provided by Xinjiang Urumqi Zhengda Feed Co., Ltd. (Urumqi, China) was fed to the calves from day 4. The study was conducted between April and May 2020 at the Shu Rui Farm (Shihezi, China). The calves were maintained according to the standards for the professional guidance process for feeding and management of the Shu Rui farm. The health of the calves was monitored and recorded after birth and throughout the experimental period.

### Sample Collection

Blood samples were obtained from six calves before morning feeding on day 21, 10 mL of blood being collected from the jugular vein of each calf with an anticoagulant vacuum blood collection tube, during the course of 30 min. The serum was centrifuged at 3,000 rpm for 15 min, separated into a centrifuge tube, and stored in the frozen state at –20°C until needed for testing.

Samples of intestinal tissue, mucosa, and contents (mid duodenum, mid jejunum, mid ileum, mid cecum, mid colon, and rectum) were obtained from different intestinal regions of the same six calves on day 21 of the experiment. Next, the 18 calves (6 in each group) were humanely sacrificed by injecting 4% sodium pentobarbital solution. Intestinal tissues were stored in 4% paraformaldehyde fixative (Biosharp, China). Samples of the mucosa and intestinal contents were stored in 50 mL cryotubes, and divided into three 2 mL sterile, enzyme-free cryotubes, which were immediately placed in liquid nitrogen for rapid freezing and stored at –80°C until needed for further analyses. All experimental steps were aseptically conducted and all intestinal tissues and digests were obtained within 20 min after euthanasia (Roth et al., 2009).

### Bacterial 16S rRNA and Fungal Internal Transcribed Spacer Gene High-Throughput Sequencing

Total gut microbial genomic DNA was extracted, wherein the primer sequences as well as PCR conditions used for the amplification of bacterial and fungal DNA were in accordance with those described in a previous study of ours (Wu et al., 2021a). According to the standard protocol of Majorbio Bio-Pharm Technology Co., Ltd. (Shanghai, China), paired-end sequencing was performed on the Illumina MiSeq PE300 platform/NovaSeq PE250 platform (Illumina, San Diego, United States). The sequences were submitted to GenBank under accession number SRP329437.

### Processing of Sequence Data

Key steps involved in sequencing data analysis are as follows. First, in order to obtain clean readings by eliminating adapter contamination and low quality data, the data was preprocessed to connect overlapping double-ended (COPE) software (V1.2.1) to obtain clean double-ended readings incorporated into labels (Liu et al., 2012). Bacterial tags were classified as operational taxonomic units (OTUs) based on 97% sequence similarity via Mothur (v1.31.2) softwares (Schloss et al., 2009). Bacterial sequences representative of OTUs was classified using a Mothur software script based on the Ribosomal Database Project (RDP) database (Cole et al., 2009). Fungal tags were clustered into OTUs based on 97% sequence similarity, using USEARCH (v7.0.1090) software (Edgar, 2013). The RDP classifier (v.2.2), based on the UNITE database, was used to classify fungal OTU representative sequences (Abarenkov et al., 2010). Mothur (v1.31.2) was used to calculate the Chao 1 index, the Shannon index, and the Simpson index, and R (v3.0.3) software was used to draw a sparse curve. QIIME (v1.80) Principal coordinate analysis (PCoA) software was used to plot beta diversity, using weighted UniFrac distance.

### Analysis of Small Intestine Immune Function by ELISA

The double-antigen sandwich method was used to determine immunoglobulin M (IgM), immunoglobulin A (IgA), immunoglobulin G (IgG), tumor necrosis factor-α (TNF-α), and interleukin-2 in calf serum (IL-2). Interleukin-1β (IL-lβ), interleukin-4 (IL-4), and secretory immunoglobulin (SIgA) of small intestine contents were determined using a biochemical kit. ELISA kits and biochemical kits were purchased from Shanghai Enzyme Link Biotechnology Co., Ltd. All tests were performed according to the manufacturer’s instructions.

### Real-Time Quantitative PCR

Tissue preparation for mRNA quantification was recently described by Schäff et al. (2018). The optical density measured at 260:280 using a spectrophotometer (NanoPhotometer, Implen GmbH, Munich, Germany) was used to estimate the quantity and quality of total RNA. For cDNA synthesis, 750 ng RNA used 200 U reverse transcriptase MMLV-RT RNase (H-) point mutant (Promega, Madison, WI) and 250 pmol random hexamer primers (Metabion International AG, Planegg-Steinkirchen, Germany) Dilute the cDNA 1:4 with diethyl pyrocarbonate water and store aliquots at –80°C. Specific primers were used to measure the mRNA expression of *interleukin-1*β (*IL-1*β), *interleukin-2* (*IL-2*), *tumor necrosis factor alpha* (*TNF-α*), *Toll-like receptors* (*TLRs*), *nuclear factor kappa-B* (*NF-κB*), *zona occludens-1* (*ZO-1*), *occludin*, *claudin*, and the copy number of 16S rRNA genes of total bacteria, *L. acidophilus, B. subtilis*, *S. cerevisiae*, and *E. coli* K99 in the small intestinal mucosa (duodenum, jejunum and ileum). Primers were designed using Primer 3 version 0.4.0 (Untergasser et al., 2012) or via literature surveys as shown (Supplementary Table 2). β-actin was used as a reference gene to normalize data. Data are presented as the ratio between the copy numbers of relevant genes and the abundance of reference genes. Relative expression of the target genes was determined using the 2-△△ Ct method.

### Short Chain Fatty Acids Concentration

A sample (100 mg) of jejunum contents was weighed, transferred to a 5 mL tube (containing 25% phosphoric acid), and vortexed vigorously until completely dissolved (4:1; v:v). As described in Bi et al. (2021), the concentration of acetate, propionate, butyrate, isobutyrate, isovalerate, and valerate was measured by gas chromatography. The concentration of Short Chain Fatty Acids (SCFA) in the jejunum is expressed as μg/g.

### Analysis of the Metaproteome in Jejunum Contents

Fecal contents in the jejunum samples were enriched with microbial cells via differential centrifugation, according to the method reported by Tanca et al. (2017). The bicinchoninic acid (Beyotime, Shanghai) method was used to quantitatively extract the protein in the jejunum sample, and SDS polyacrylamide gel electrophoresis was used to evaluate the quality of the protein extract in the sample. The protein sample (100 μg) was digested by the FASP method (Wiśniewski et al., 2009). After enzymolysis, similar amounts from each biological sample were removed and mixed. Of this, 100 μg were classified via high pH RP reversed-phase chromatography (chromatograph: Agilent 1100 (including Chemstation, 214 nm dad detector, and vacuum degasser).

The column was Waters XBridge C18 (5 μm, 4.6 × 250 mm, 120 Å). Protein Discoverer 2.1.0182 (Thermo Fisher Scientific, Rockford, IL, United States) was used for protein retrieval and analysis. Biological samples were collected for data-independent acquisition (DIA) and analyzed quantitatively using Skyline software (Department of Genome Sciences, University of Washington, Seattle, WA) (Egertson et al., 2015).

The sequences were submitted to MassIVE and can be downloaded via the following address: fttp://massive.ucsd.edu/MSV000087975/.

### Liquid Chromatography-MS/MS Analysis

Jejunum content samples were analyzed using an online nanospray Orbitrap Fusion Lumos Tribrid mass spectrometer (Thermo Fisher Scientific, MA, United States) with an EASY-nLC system (Thermo Fisher Scientific, MA, United States). The peptides were dissolved in solvent A (0.1% formic acid in water) spiked with 1× iRT standard (iRT Kit; Biognosys, Schlieren, Switzerland). Exactly 1 μg of peptide sample was loaded onto an Acclaim PepMap C18 column (75 μm × 25 cm) and separated using 120-min linear gradient. Column flow rate was maintained at 400 nL/min, while column temperature was maintained at 40°C. An electrospray voltage of 2100 V was applied. A full scan was performed at m/z 350–1,200 with a resolution of 120,000 at m/z = 200, and maximum injection time of 50 ms. The MS/MS scan was performed with higher-energy collision-activated dissociation for m/z 200–2,000 with a resolution of 30,000 at m/z = 200, and maximum injection time of 90 ms. Collision energy was 32%, and the stepped collision energy was 5%. DIA was performed with 25 variable isolation windows with a 1 Da overlap and total cycle time of 3 s.

The DDA mode was used to construct a spectral library for protein identification and quantification using DIA. One microgram of peptides from each sample was combined, and the mixture was redissolved in 50 μL of buffer C (20 mM ammonium formate in water, pH = 10.0, adjusted by ammonium hydroxide). Next, the combined peptide solution was subjected to high-pH reversed-phase Liquid Chromatography (LC) fractionation via an Ultimate 3000 system (Thermo Fisher Scientific, MA, United States) with a C18 column (4.6 mm × 250 mm, 5 μm). Column flow rate was maintained at 1 mL/min, while column temperature was maintained at 40°C. The fractions were continuously collected. Each fraction was dried in a vacuum-freeze dryer, redissolved in 50 μL of solvent A (0.1% formic acid in water), and subjected to LC-MS/MS analysis; the injection volume was 5 μL. Dynamic exclusion was enabled within a duration of 30 s. An MS/MS scan was performed with 1.6 Da isolation window widths. The other MS parameters, LC gradient conditions, and LC column were similar to those used in the DIA experiments.

### Microbial Function Analysis

All quantifiable microbial protein sequences were annotated using the Clusters of Orthologous Genes (COG) database (version 2014), as previously described (Zhang et al., 2016). KEGG ortholog (KO) annotation of protein sequences was conducted using the GhostKOALA web application (Kanehisa et al., 2016). Taxonomic assignment of the proteins was performed using MEGAN 6 (Huson et al., 2007). The taxonomy of a protein group was assigned using the lowest common ancestor (LCA) of all proteins within that protein group.

Pathway enrichment analysis was performed using STRING (version 10.5) (Szklarczyk et al., 2017). Protein interaction networks were exported from STRING and visualized using Cytoscape software (version 3.4.0).

### Statistical Analysis

The Durbin Watson test was used to examine the randomness of initial weight data and the effectiveness of randomization. The GLIMMIX program SAS 9.4 was used to analyze serum immunoglobulin, SCFA concentration, SIgA and fecal microbial data on the basis of repeated measurement and compound symmetrical variance and covariance structure. The fixed effect of treatment, day, the interaction between treatment and day, and the random effect of calf identity were included in the repeated measurement model. A melting curve analysis was generated to verify the specificity of the reaction after each quantitative real-time PCR analysis. Select housekeeping gene β-Actin was used as a reference gene to normalize the mRNA expression of the target gene. The gene expression data of replicated samples were calculated using the CT method (Pfaffl, 2001). The relative expression of target genes in group C was set to 1.0. Each sample was measured in triplicate. The data are expressed as the least squares mean and the standard error of the mean. Tukey’s multi range test was used to evaluate the differences between the treatment groups.

DDA data were analyzed using Proteome Discoverer 2.1.0182 (Thermo Fisher Scientific, Rockford, IL, United States). MS1 tolerance was set to 10 ppm, and MS/MS tolerance was 0.02 Da. All DDA MS/MS spectra were searched against the database of bacterial proteomes downloaded from the UniProt database (23,730,617 protein entries,^1^ access date July 2019). The Bacteria, Eukaryota, and Archaea databases in the Uniprot database were “searched and compared.” The FDR cut-off for PSM, peptide, and protein group levels was 1%. Raw DIA data were then processed and analyzed via Skyline (Department of Genome Sciences, University of Washington, Seattle, WA, United States) using the default settings. The top three filtered peptides that passed the 1% *Q*-value cut-off were used to calculate the major group quantities.

Annotation of identified proteins was performed using KOBAS^2^ in which several databases (i.e., GO, KEGG, and eggNOG) were integrated. Differentially expressed proteins were analyzed using mapDIA, a software package used to preprocess and statistically analyze quantitative proteomics data. The abundance of each phylum was calculated by summing the intensities of all proteins corresponding to that phylum. Data visualization was conducted using R packages, including heatmaps, circles, and ggplot2.

## Results

### Gut Microbial Diversity

Our data indicated that bacterial amplicon sequencing of the intestinal content microbiota generated 6,978,315 high-quality sequences that were assigned to a total of 33952 OTUs based on a 97% nucleotide sequence similarity, with an average of 32,112 sequences per sample (21,230–35,090 sequences). PCoA (Supplementary Figure 2) and analysis of similarities (ANOSIM) analyses (Supplementary Table 3) revealed that supplementing *E. coli* infected neonatal calves with MSP induced significant differences in the microbial structure of different gut region digesta-attached microbiota. The diversity of microbiota in the D and P groups was similar to that of control (C) on day 21, whereas the duodenal, ileal and jejunal microbiota of the D and P groups demonstrated a marked shift along principal component 1 compared with that of the C group.

**FIGURE 1:**
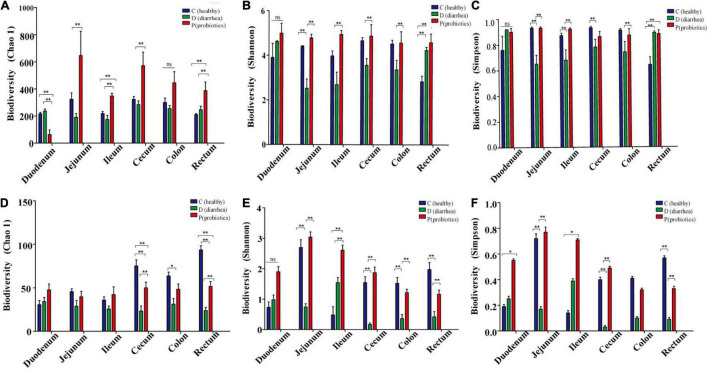
The abundance and diversity index of microorganisms in different intestinal contents of Holstein calves. **(A–F)** Represent the abundance of Chao1, Shannon and Simpson and the diversity of bacterial and fungal microflora diversity indexes. *P < 0.05, **P < 0.01.

Bacterial diversity on day 21, which was estimated using Chao1, Shannon, and Simpson indices, was lowest in the jejunum, ileum, cecum, and colon of the D group, as compared to those of the H and P groups. In addition, the Chao1 and Shannon indices for the C group and P group were all significantly higher than those for the D group. There were no significant differences between the diversities in the duodenums of the C and D groups (Figure 1 and; Supplementary Table 4).

The fungal diversity index, Chao 1, for the cecum, colon, and rectum of the D group was significantly lower than that for the C and P groups (*P* < 0.01; *P* = 0.02; and *P* < 0.01, respectively). The Shannon diversity index for the jejunum, ileum, cecum, and rectum of the D group was significantly lower than that of the C and P groups (*P* < 0.01; *P* = 0.01; *P* < 0.01; and *P* < 0.01, respectively). There were no significant differences between the C and P groups. The Simpson index of fungal diversity for the jejunum, cecum, and rectum of group D was significantly lower than that of the C and P groups (*P* < 0.01; *P* < 0.01; and *P* < 0.01, respectively) (Figure 1 and Supplementary Table 4). In addition, the results indicated that treatment and gut segment had a significant effect on the α-diversity of bacteria and fungi.

### Relative Abundance of Bacterial and Fungal Taxa

**FIGURE 2:**
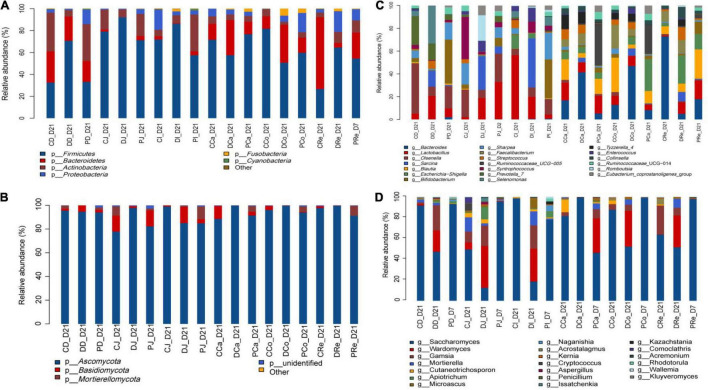
Composition of the different gut region digesta-associated microbial community. **(A,C)** Show the composition of the microbial community in the three groups (C, D, and P) with respect to bacterial phyla and genera. **(B,D)** Show the composition of the microbial community in the three groups (C, D, and P) with respect to fungal phyla and genera (CD, DD, and PD represent groups C, D, P dudenum; CJ, DJ, and PJ, represent groups C, D, P jejunum; CI, DI, and PI represent groups C, D, P ileum; CCa, DCa, and PCa, represent groups C, D, P cecum; CCo, DCo, and PCo, represent groups C, D, P colon; CRe, Dre and PRe, represent groups C, D, P rectum).

*Firmicutes*, *Actinobacteria*, and *Bacteroidetes* were the dominant bacterial phyla in the duodenum, jejunum, ileum, cecum, colon, and rectum, followed by *Proteobacteria* and *Fusobacteria*. The relative abundance of *Firmicutes* in the duodenum (*P* = 0.01) and jejunum (*P* = 0.04) of neonatal calves in the D group was higher than those in the C and P groups. The relative abundance of *Actinobacteria* in the duodenum (*P* = 0.01) and jejunum (*P* = 0.03) of calves in the D group was lower than that in the C and P groups, whereas in the ileum (*P* = 0.01) it was higher than that in the C and P groups. The relative abundance of *Bacteroidetes* in the jejunum (*P* = 0.03) of calves in the D group was lower than that in the C and P groups, while no significant differences were observed between other intestinal segments (Figure 2 and Supplementary Table 5).

The top 20 genera at the genus level, were detected and further analyzed: *Olsenella*, *Prevotella_*7, *Selenomonas*, *Bifidobacterium*, *Lactobacillus*, *Blautia*, *Faecalibacterium*, *Syntrophococcus*, and *Ruminococcaceae*_UCG-005 were the predominant bacterial genera in the duodenum and jejunum. The relative abundances of *Sarcina* (*P* < 0.01), *Streptococcus* (*P* < 0.01), *Selenomonas* (*P* < 0.01), and *Lactobacillus* (*P* < 0.01) in the duodenums of the D group were significantly higher than those of the C and P groups.

However, the relative abundance of *Bifidobacterium* in the duodenum (*P* = 0.02), jejunum (*P* = 0.02), and ileum (*P* = 0.01) of the P group was significantly higher than that of the C and D groups. The relative abundance of *Sharpea* in the jejunum (*P* = 0.04) of the D group was significantly lower than that of the C and P groups (*P* = 0.02), while the relative abundance of *Prevotella*_7 in the jejunum of the P group was significantly higher than that of the C and D groups. No significant differences were observed between other gut regions. In addition, no significant difference was observed between the hindguts of the three groups (Figure 2 and Supplementary Table 6).

The microbial composition of the calf gut indicated that Ascomycota was the dominant phylum followed by *Basidiomycota* and *Mucoromycota* (Figure 2 and Supplementary Table 6). A trend toward greater relative abundance of *Ascomycota* was observed in MSP supplemented neonatal calves compared to that in the C and D groups (*P* < 0.10). However, the relative abundance of *Mucoromycota* in the colon of group D neonatal calves was higher than that in the group C and P neonatal calves (*P* = 0.03). In addition, the relative abundance of *Basidiomycota* in the C group was significantly higher than that in the other two groups (*P* = 0.01).

Calf gut microbial composition indicated that Saccharomyces, Aspergillus, Wardomyces, and Acaulium were the predominant genera, followed by Cladosporium, Filobasidium, Geotrichum, Mortierella, Kazachstania, Kernia, Cutaneotrichosporon, and Scopulariopsis (Figure 2 and Supplementary Table 6). The relative abundances of Acaulium in the duodenum, jejunum, ileum, and cecum of the D group tended to be higher than those of the C and P groups (P = 0.01; P < 0.01; P < 0.01; and P = 0.01, respectively). The relative abundance of Saccharomyces in the duodenum, jejunum, and ileum of the P group was significantly higher than that of the C and D groups (P < 0.01; P < 0.01; and P = 0.03, respectively). The relative abundance of Wardomyces in the jejunum, ileum, cecum, colon, and rectum of group D was significantly higher than that of the C and P groups (P < 0.01; P = 0.02; P = 0.03; P = 0.02; and P = 0.02, respectively). The relative abundances of Kazachstania and Acremonium in group P were significantly higher than those in the C and D groups (P < 0.01). The relative abundance of Cladosporium and Pichia in the jejunum of group D was significantly higher than that of the C and P groups (P < 0.01). In addition, the relative abundance of fungi at the phylum and genus levels was significantly different in the different treatment groups of neonatal calves infected with E. coli K99.

### Immune Function

MSP supplementation caused significant differences in the levels of IgM and TNF-α in the sera of neonatal calves infected with *E. coli* K99 (*P* < 0.05), wherein the C group differed significantly from the P group (*P* < 0.01), while the calf sera of the C, D, and P groups showed significant differences. The concentrations of IgG, IgA, IL-1, IL-2, and IL-4 were not significantly affected (*P* > 0.05) (Table 1).

**TABLE 1:** Effect of MSP supplementation on the serum immune function and the content of SIgA in neonatal calves intestinal mucosa of neonatal calves infected by *E. coli* K99.

Item	Treatment^1^				*P*-value
	C	D	P	SEM	
IgM (μg/mL)	2780.1^a^	2058.21^b^	2234.47^b^	113.40	0.01
IgG (mg/mL)	5.84	6.23	6.68	0.25	0.44
IgA (μg/mL)	1533.56	1388.96	1318.10	43.41	0.11
IL-1 (pg/mL)	376.11	354.41	331.84	9.94	0.19
IL-2 (pg/mL)	430.22	440.38	420.70	11.86	0.81
IL-4 (pg/mL)	38.91	39.98	40.92	0.66	0.49
TNF-α (pg/mL)	133.55^a^	130.81	116.58	3.07	0.04
SIgA (μg/g)					
Duodenum	447.78^a^	297*^c^*	360.16^b^	18.25	<0.01
Jejunum	431.76^a^	281.84*^c^*	368.32^a^	18.49	<0.01
Ileum	419.61^a^	278.17^b^	364.68^a^	19.56	<0.01

*^a,b^Means in the same row with different superscripts are significantly different (P ≤ 0.05).*

*^1^C, Control group; D, Diarrhea group; P, MSP group; respectively.*

The effect of MSP supplementation on SIgA in the duodenum, jejunum, and ileum mucosa of neonatal calves infected with *E. coli* K99 was significantly different from that of the D and P groups (*P* < 0.01). In addition, SIgA concentrations in the duodenum, jejunum, and ileum mucosa of calves in group P were significantly higher than those in group D (*P* < 0.01). The SIgA concentrations in the duodenum and jejunum mucosa of calves in the C group were significantly higher than those in the D and P groups (*P* < 0.01) (Table 1).

### Expression of Inflammation-Related and Intestinal Barrier-Related Genes

**FIGURE 3:**
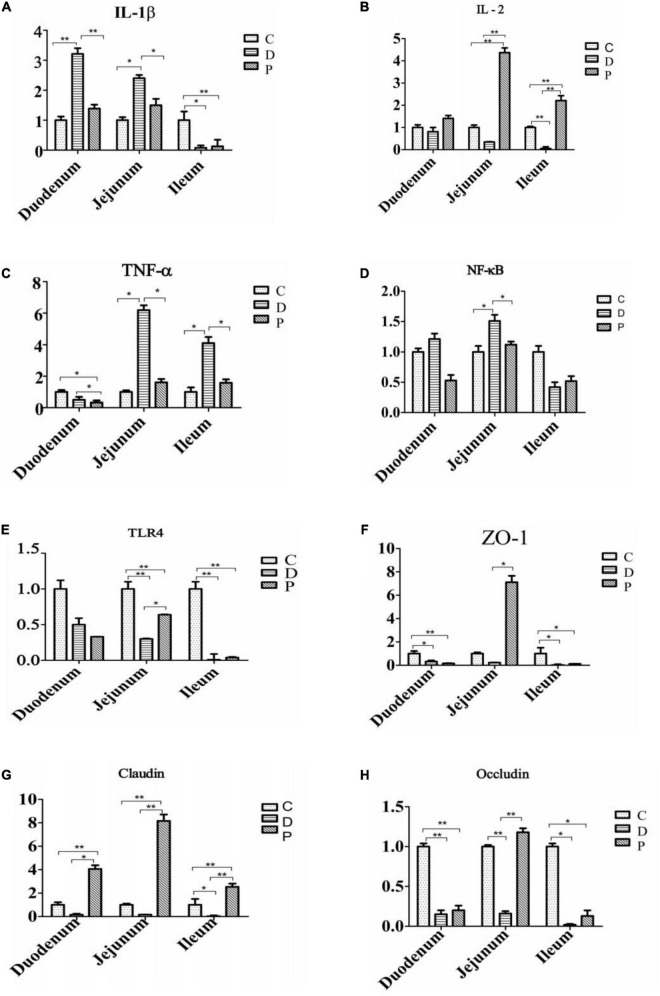
Effect of supplementation with MSP on the relative expression of small intestinal mucosal immune factors and tight junction protein mRNA in neonatal calves infected by *E. coli* K99 (C, Control group; D, Diarrhea group; P, MSP group; respectively). **(A)** Interleukin-1β, *IL*-1β. **(B)** Interleukin-2, *IL-2*. **(C)** Tumor necrosis factor-α, *TNF-α*. **(D)** Nuclear factor kappa-B, *NF*-κ*B*. **(E)** Toll-like receptor 4,TLR4. **(F)** Zonula occludens 1, *ZO-1*. **(G)** Claudin. **(H)** Occludin. *P < 0.05, **P < 0.01.

The relative expression of *IL-1*β, *NF-κB*, and *TNF-α* mRNA in the duodenal and jejunal mucosa of the D group was significantly higher than those of the C and P groups (P < 0.01). The relative expression of *IL-2* mRNA in the jejunum of calves in the P group was significantly higher than that in the D group (*P* < 0.01). The relative expression of *ZO-1* mRNA in the jejunum mucosa of calves in group P was significantly higher than that in groups C and D (*P* < 0.01). The relative expression of *claudin* mRNA in the mucosa of calves in the duodenum, jejunum, and ileum of the P group was significantly higher. The expression of *occludin* mRNA was significantly higher in the P group than in the C and D groups (*P* < 0.01), while the relative expression of *occludin* mRNA in the jejunum and ileum mucosa of calves in group P was significantly lower than that in groups C and D (*P* < 0.01) (Figure 3).

### Short Chain Fatty Acid Concentration in the Jejunum of Neonatal Calves

To further analyze microbial activity in jejunum contents, we measured the concentrations of short-chain fatty acids (SCFAs), using them as a proxy reflecting microbial metabolic activity in our gut samples. The concentrations of acetate, propionate, butyrate, and isovaleric acid were significantly higher than those in the C and P groups (Table 2). The concentration of total SCFA in the P group was increased compared to that in the D group, while being significantly higher in the C group than that in the D group (Table 2). In addition, there were significant differences between the concentrations of acetic acid, propionic acid, butyric, isobutyric, isovaleric, and isocaproic acid contents among the three groups.

**TABLE 2:** Short chain fatty acid (SCFA) concentration and molar proportion in the jejunum contents of neonatal calves.

	Treatment^1^				*P*-value
SCFA (μg/g)	C	D	P	MSE	
Acetic acid	312.69^b^	436.56^b^	641.57^a^	42.57	<0.01
Propanoic acid	36.91^b^	60.92^b^	153.36^a^	19.37	0.02
Butanoic acid	736.88^a^	58.73^b^	182.97^b^	12.4	0.03
Isobutyric acid	3.88	3.89	7.29	0.98	0.24
Valeric acid	20.59	20.80	79.69	8.72	0.06
Isovaleric acid	1.76^b^	3.86^b^	47.98^a^	5.5	0.02
Hexanoic acid	1798.44^a^	142.91^b^	275.89^b^	267.41	0.01
Isohexanoic acid	1.76^a^	0.00^b^	0.00^b^	0.31	0.02
Total acid	2312.91^a^	972.59^b^	1595.47^ab^	81.05	0.04

*^a,b^Means in the same row with different superscripts are significantly different (P ≤ 0.05).*

*^1^C, Control group; D, Diarrhea group; P, MSP group; respectively.*

### Metaproteomics Profiling of Jejunum Content

An advantage associated with metaproteomics is that the microbial population as well as host proteins can be measured in a single analysis. Therefore, we further analyzed microbial function in the calf jejunum, via a metaproteomics analysis aimed at detecting microbial and host proteins in our samples.

We quantified 22,080 unique peptides and 4,414 proteomes in nine samples, with an average recognition rate of 39 ± 8% (mean ± standard deviation). On average, 2,453 ± 89 unique peptides and 735 ± 33 proteomes were identified for each sample. Among the 4,414 proteomes, 3,679 proteins (83%) were from host calves, and only 735 (17%) were from calf jejunum-content microorganisms. However, host proteins accounted for 5.54 times the intensity of the total proteins. We used macroproteomics to characterize the microbiomes of 9 jejunum samples from groups C, D, and P (three samples in each group). The results indicated that 226 showed expressions specific for each group.

**FIGURE 4:**
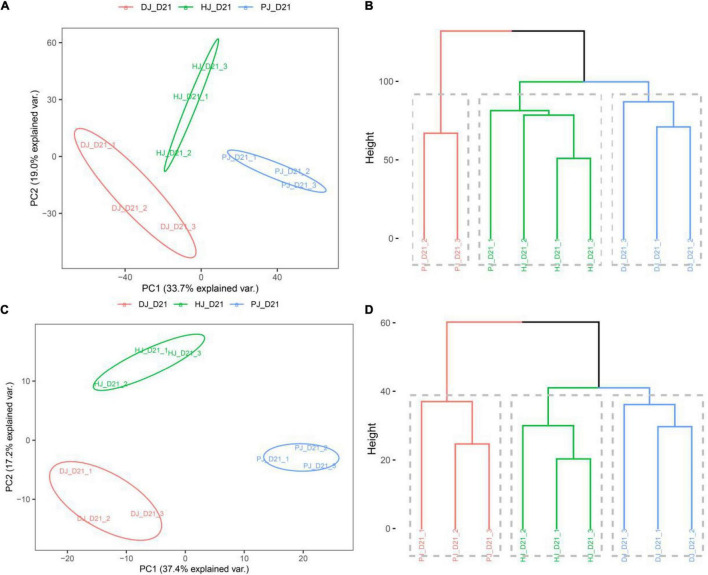
Host and microbiota proteome landscape alterations in neonatal calves infected by *E. coli* K99. **(A,C)** PCA score plot of calf-derived and microbiome-derived proteins quantified in ≥ 50% of the samples; **(B,D)** group mean clustering based on Mahalanobis distances calculated with MANOVA test using the first 10 PCs of PCA on calf-derives proteins, respectively.

Data obtained via further analyses of the gut microbiome function in the jejunum indicated that probiotics caused shifts in intestinal microbiome function, as evidenced by the scatterplot generated from the principal component analysis (PCA) and the Cluster Dendrogram (Figure 4). PCA was performed using calf-derived proteins or non-calf proteins (i.e., microbiota-derived) quantified in ≥ 50% of the samples. The PCA score plot for calf proteins showed an obvious separation among the three groups (Figure 4A). Furthermore, PCA analysis of microbiota-derived proteins also showed obvious separation among the three groups (Figure 4C).

Multivariate analysis of variance (MANOVA) of calf-derived proteins yielded greater differences, revealing significant differences (*P* < 0.01) between groups C and D (Figure 4B). By contrast, MANOVA of microbiota-derived proteins revealed significant differences between C, D, and P groups (Figure 4D). To identify treatment-related alterations in host and microbiota, we combined samples from C, D, and P in jejunal function recovery for further analysis.

### Elevated Microbial Response to Carbohydrates and Defense Mechanisms in Neonatal Calves

**FIGURE 5:**
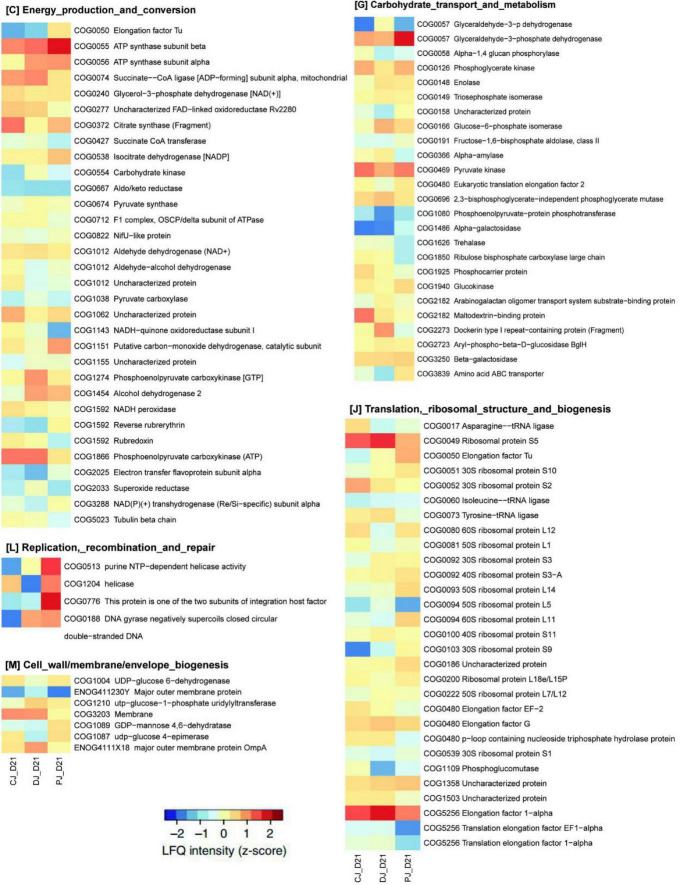
Functional compositions of microbiome in neonatal calves infected by *E. coli* K99. Heatmap of differentially abundant COGs in neonatal calves. Representative COG categories are shown, and the colors indicate the average label free quantitation (LFQ) intensity for each subgroup of samples. Each row corresponds to a COG with the COG ID and the name indicated.

**FIGURE 6:**
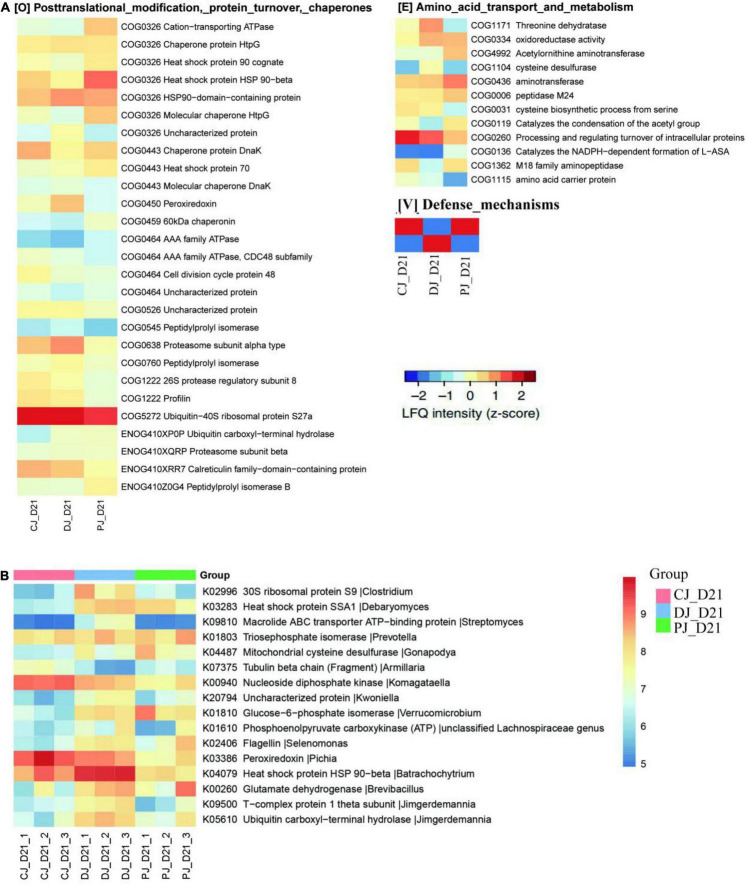
Functional compositions of the microbiome in neonatal calves infected by *E. coli* K99. **(A)** Heatmap of differentially abundant COGs in neonatal calves. Representative COG categories are shown, and the colors indicate the average LFQ intensity for each subgroup of samples. Each row corresponds to a COG with the COG ID and name indicated. **(B)** Heatmap shows the average LFQ intensity for each group of samples. Each row represents one protein group, and the corresponding KO ID, KO name and taxonomic assignment are shown in the right panel.

To assess microbial function, we annotated all quantified microbial proteins using the COG database and obtained 330 COGs from 24 COG categories. A total of 154 COGs were identified in all three groups (Figures 5, 6).

**FIGURE 7:**
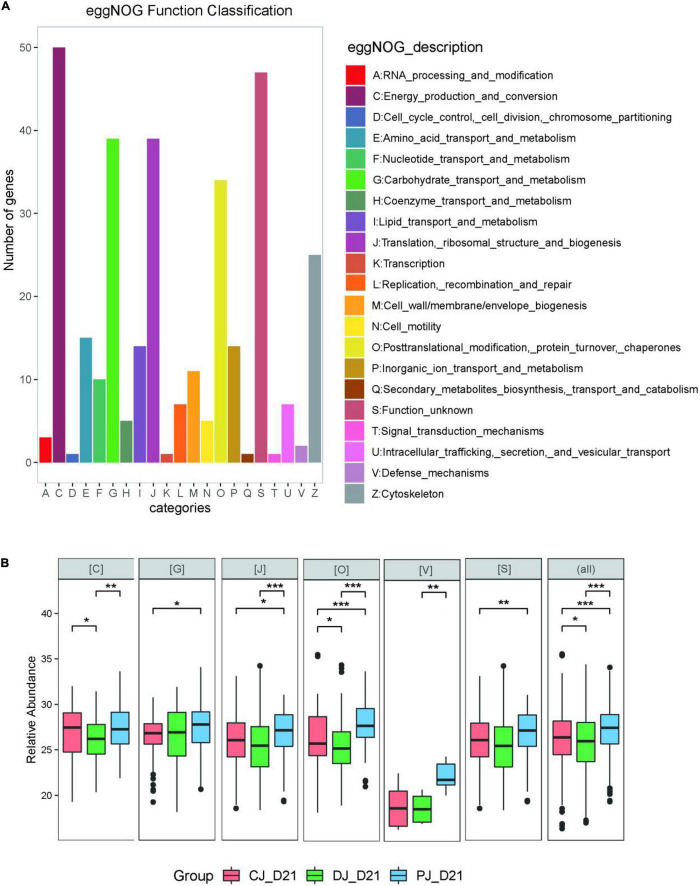
**(A)** eggNOG classifications of the neonatal calves gut Metaproteomics. **(B)** LFQ intensity of COG category C (Energy production and conversion), category G (Carbohydrate transport and metabolism), category J (Translation, ribosomal structure and biogenesis), category O (Posttranslational modification, protein turnover, chaperones), and category V (Defense mechanisms) in microbiome of neonatal calves infected by *E. coli* K99. For the box plot, the bottom and top of the box represent the first and third quartiles, respectively. The middle line represents the sample median. Whiskers are drawn from the ends of the IQR to the furthest observations within 1.5 times the IQR range. Outliers > 1.5 times the IQR are indicated with circle. *p < 0.05, **p < 0.01, ***p < 0.001.

At the COG category level, we found that category G (carbohydrate transport and metabolism), category O (posttranslational modification, protein turnover, chaperones) category V (defense mechanisms), and category S (function unknown) were significantly increased in the MSP group compared to those of the C and D groups (Figure 7). Defense mechanisms in the P group, in particular, were significantly increased compared to those in the C and D groups. Two COGs belonging to defense mechanisms were identified as differentially abundant in the P group microbiome compared to that in the C and D groups (Figure 7). KO annotation identified 516 KOs corresponding to 735 proteins. The Wilcoxon rank-sum test identified 116 proteins that were significantly different among the three groups, among which six were upregulated in the D group and downregulated in the C and P groups, where ATP-binding cassette (ABC) transporters (KO:K09810), ubiquitin carboxyl-terminal hydrolases (UCHs)(KO:K05610), Glucose-6-phosphate isomerase (KO:K01810), 30S ribosomal protein S9 (A0A099S8L6), and heat shock protein 90 OS (KO:K04079) were significantly upregulated in group D compared with groups C and P (Figure 6B). Interestingly, dietary supplementation with MSP only decreased the expression of both HSPs, where the difference was significant in the case of HSP90.

We identified 666 microbial species (666 ± 27 species per sample) with a minimum of one distinctive peptide from three different kingdoms (bacteria, fungi, and archaea). Linear discriminant analysis effect size (LEfSe) identified seven phyla and 71 species as differentially abundant among the three groups. All seven differentially abundant phyla, namely *Proteobacteria*, *Firmicutes*, Chytridiomycota, Ascomycota, and Basidiomycota, were decreased in group P, compared to those in D, while Blastocladiomycota, Zoopagomycota, and Crenarchaeota, were increased compared to the D and C groups. Of the four most significantly changed strains in the D group, *Prevotella* and *Streptomyces*, were decreased and *Pichia* and *Selenomonas* were increased, respectively, compared to C and P groups. This finding agreed with those of a 16S rRNA and ITS sequencing study using a similar type of calves. However, in contrast to the 16s rRNA and internal transcribed spacer (ITS) sequencing study using a similar type of calves, *Batrachochytrium* in the D group was higher than that in the C and P groups. Previous studies have indicated that genera identified via metagenomics may differ from those identified via the 16S rRNA and ITS sequencing (Walker and Lue, 2015; Hubert et al., 2017; Hu et al., 2018).

### Host-Protein in Jejunum of Neonatal Calves

To identify calf proteins that were differentially abundant in group D compared to groups C or P, or in groups D or P compared to C, a Venn diagram was conducted for each pair of groups. We identified 55, 57 and 52 differentially abundant calf proteins for C vs. D, P vs. D, and P vs. C, respectively (Supplementary Figure 2 and Supplementary Table 7). Among the differentially abundant calf proteins, 11 were upregulated and 12 were downregulated in the other groups compared with group D. The proteins, Q3ZBF3, P62248, Q9XSC9, and Q3ZC02, in group D were significantly downregulated compared to those in C, and the proteins, Q3ZBF3, Q3ZC02, and Q9XSC9, in group P were significantly upregulated compared to those in group D (Supplementary Table 7).

Gene ontology enrichment analysis of differentially abundant calf proteins in P and D groups demonstrated that the most highly enriched biological functions involved metabolic and cellular processes (Supplementary Figure 4 and Supplementary Table 8).

To gain insights into host-microbiome interactions, we performed co-occurrence analysis for all identified differentially abundant calf proteins and microbial functions and found that both calf proteins and microbial functions were substantially associated with each other (Supplementary Figure 4). In the protein interaction networks (Supplementary Figure 4 and Supplementary Table 9), the microbial heat stress protein, HSPCOG 0326, which displayed the highest association (*n* = 5) with calf proteins, acted as an important microbial hub connecting the host and microbiome nodes, thereby suggesting a “hub” role for these proteins. As mentioned above, 54 of the 154 significantly increased microbial COGs belonged to categories G, O, and V, which were related to energy production and conversion and DNA damage/mismatch responses, indicating that in calves, microbes are utilizing a considerable amount of energy production and conversion as well as defensive mechanisms against stressors derived by the host.

HSP90 is associated with five host proteins, and generates an inflammatory response via the necroptosis pathway, thereby facilitating the upregulation of IL-1β expression (KO4079). The microbial host-protein interaction map indicated that HSP90 was associated with the host protein, Q3MHN2, which participates in the immune system process and responds to stimuli affecting the host. Q3MHN2 contains a membrane attack complex (MAC)/perforin domain, and the MAC of the complement system is a well-characterized innate immune effector (Merselis et al., 2021). These findings proved that the relationship between neonatal calves infected by *E. coli* K99 and *Batrachochytrium salamandrivorans* may be at the strain level instead of at the species level, and that its sub-species level dysbiosis may be related to differences between immune functions in response to oxidative stress.

## Discussion

The diversity of intestinal microbiota showed a gradually increasing trend during the growth of newborn calves. Shannon diversity differs between animals of different ages (Alipour et al., 2018; Dias et al., 2018). The establishment of intestinal microflora in calves within 7 weeks of birth is closely related to calf health and growth (Oikonomou et al., 2013). Beta diversity decreased with age, while alpha diversity increasing (Dill-McFarland et al., 2019; Li et al., 2020). In addition, the Shannon and Chao1 indices showed significant changes in diversity from birth until the 8th week. Previous studies on dairy cows have shown that the Chao1 index gradually increases with body weight over time from the 1st to the 7th week before weaning. Older calves have a higher Chao1 index (Oikonomou et al., 2013). Diarrhea or an infection may cause a decrease in microbial diversity (Kim et al., 2021; Xin et al., 2021). While most previous studies focused on investigating and interpreting calf manure samples, only a relatively few studies, such as ours, have focused on investigating the effects of feeding calves with compound probiotics on the intestinal microflora of these animals. The results of our current study on the diversity of microbiota in calf fecal samples were consistent with those of previous studies (Li et al., 2018, Dill-McFarland et al., 2019). Our results showed that the Chao1 and Shannon indices of the fecal microbiota gradually changed over time. However, the diversity of microbiota in the small intestine of D calves was higher than that of the fungi, while Chao1 and Shannon indices were lower than those of the C and P groups, in addition to which α-diversity was significantly reduced. Thus, intestinal microbiota diversity of calves infected with *E. coli* can be significantly improved by adding compound probiotics.

However, calves have a large amount of mucosal-associated *E. coli* in their intestines in the first week after birth, which causes them to be more likely infected with pathogens. Therefore, reducing pathogen colonization and intestinal infections has become an effective way to improve intestinal health (Song et al., 2018). This study shows that *Streptococcus* and *Enterococcus* can use the available oxygen in the intestine and create an anaerobic environment, which is beneficial to the colonization of strict anaerobic intestinal residents such as *Bifidobacterium* and *Bacteroides* (Conroy et al., 2009). However, recent studies have indicated that gut microbiota possibly begin colonizing during the birth process (Malmuthuge et al., 2015; Song et al., 2018). The transmission process of maternal microbiota to newborn calves may originate at the amniotic fluid stage, in a manner similar to that reported for NB human infant gut microbiota (DiGiulio, 2012) and meconium (Moles et al., 2013). Significant individual variations observed among the NB calves may be due to variability in the transmission process from the mother cow to the birth environment (uterus, vaginal canal, and fetal membranes) (Klein-Jöbstl et al., 2019). The study found that the relative abundance of *Lactobacillu* and Shigella was higher in newborn calves than in D21 calves (Song et al., 2018).

*E. coli* infection can destroy the integrity of the intestinal tract and severely damage its intestinal function, thereby disrupting the balance of intestinal microbiota and leading to a decline in immune function (Dubreuil, 2017; Braz et al., 2020). Antibiotics play a vital role in many bacterial infections (Liu et al., 2017). However, antibiotics are known to cause intestinal microbiota imbalance, affect host metabolism, and lead to chronic inflammation (Scott et al., 2018). Relevant studies have shown that probiotics have a positive effect in reducing the susceptibility of calves to intestinal infections before weaning (Markowiak and Śliżewska, 2018; Mazloom et al., 2019; Plaza-Diaz et al., 2019). The anti-diarrheal function of MSP may be associated with its role in immunity (Inatomi et al., 2017; Villot et al., 2020). Concentrations of circulating immunoglobulins, especially those of IgG, IgM, and IgA, are important indicators of immune function (Crassini et al., 2018). Some studies have indicated that supplementation with probiotics promotes the immune responsiveness of calves by improving serum IgG concentration (Song et al., 2019). Other studies have shown that supplementing with probiotics can reduce the rate of calf diarrhea and increase the serum IgM concentration on the 14th day, while increasing the serum IgA, IgG and IgM concentrations on the 28 day, respectively (Wang et al., 2019).

Intestinal SIgA has become the main regulator of intestinal microbiota through agglutination and enhancement of the opportunity to eliminate pathogens (Moor et al., 2017; Guo et al., 2020, 2021). Furthermore, Karamzadeh-Dehaghani et al. (2021) demonstrated that supplementation with probiotics improved the growth performance and immune response of calves. Consistent with these findings, we found that the gut SIgA concentration in the jejunum of calves in the MSP supplemented P group was increased by 86.48 μg/g compared to that in the D group. Studies have indicated that the regulation of potential intestinal pathogens and fungal colonization may enhance immunity (Nakawesi et al., 2020; Guo et al., 2021), whereas antibiotic exposure disturbs early GM, leading to intestinal dysbiosis and reduced SIgA (Guo et al., 2021), and that supplementation with additional probiotics may help enhance normal intestinal functioning (Tanaka et al., 2017; Plaza-Diaz et al., 2019; Roggero et al., 2020). The results of our study indicated that MSP supplementation improved the diversity and structure of the small intestinal microbiota in neonatal calves infected with *E. coli* K99, and simultaneously increased the secretion of SIgA compared to that in the antibiotics group.

The gut tract, which is one of the most microbiologically active ecosystems, plays a crucial role in the functioning of the mucosal immune system (MIS). Probiotics are known to exert positive effects on intestinal diseases. Among other effects, probiotics exert barrier-modulating effects and alter the inflammatory response toward pathogens in *in vitro* and *in vivo* intestinal infection models (Lodemann et al., 2017; Wang et al., 2019; Maldonado Galdeano et al., 2019), prevent or ameliorate damage to epithelial integrity caused by pathogenic challenge (Sherman et al., 2005; Johnson-Henry et al., 2008), and modulate the ease of cytokine secretion (Bahrami et al., 2011; Paszti-Gere et al., 2012). There is a close connection between the gut microbial community and the host immune system (Duparc et al., 2017; Cani and Jordan, 2018; Cani, 2019). Several reports have validated the fact that manipulating gut microbiota using probiotics, such as *Bacillus subtilis* (Jia et al., 2021) and *Lactobacillus acidophilus* (Lépine et al., 2018) may affect host gut barrier function and gut microbiota composition (Régnier et al., 2021). A growing number of studies have shown that certain probiotics (such as *Lactobacillus acidophilus*) can regulate the obstruction of intestinal epithelial fluid absorption and secretion caused by diarrhea (Borthakur et al., 2008; Singh et al., 2014). The results of the present study showed that MSP supplementation significantly upregulated relative mRNA expression of ***ZO-1***, ***claudin-1***, and ***occludin*** in the jejunum of *E. coli*-infected neonatal calves. Among them, a downward trend was observed in group D, indicating that in group P, claudin mRNA levels were increased to reduce intestinal *E. coli* adhesion, and to protect the mechanical barrier and paracellular permeability of the small intestine.

SCFA has important physiological effects on the host (Perego et al., 2018; Chen and Vitetta, 2019). Acetic acid is a short-chain fatty acid produced by intestinal bacteria. It inhibits the growth of pathogens and regulates the metabolism and gene expression of B cells, and promotes the secretion of SIgA, thereby maintaining and repairing the intestinal morphology of animals (Garcia et al., 2007; Kim et al., 2016; Wu et al., 2017; Bourassa et al., 2018). The results of this study indicate that MSP maintains intestinal health by increasing the acetic acid content in the jejunum of newborn calves infected with *E. coli* K99. Previous studies have shown that most *Bacteroides*, *Bifidobacterium*, *Prevotella*, and *Rumenococci* produce acetic acid through “pyruvate via the acetyl-CoA pathway” (Louis et al., 2014; Koh et al., 2016). In our study, the relative abundances of *Bacteroides*, *Bifidobacterium*, *Prevotella*, and *Rumenococcus* in the calf jejunum content of the MSP group was significantly higher than those of the antibiotic group. The results of this study indicated that MSP promotes the recovery of the intestinal function of calves following *E. coli* infection. Further, the results of this study are consistent with those of previous studies (Louis et al., 2014; Koh et al., 2016; Heeney et al., 2019).

Various functions of HSPs that target microbial stimuli include maintaining protein stability and functionality, activating, potent immune responses, immunomodulation of host-microbe relationships, and acting as biomarkers (diagnosis of infection) (Bolhassani and Agi, 2019). Various functions of HSPs that may be used to control bacterial infections have been studied (Bolhassani and Agi, 2019). Studies have shown that high expression of HSP90 may lead to the secretion of inflammatory cytokines in the body (Abaza, 2014). Studies have shown that some HSPs are effective inducers of innate and adaptive immunity. In addition, HSPs exert cytoprotection against inflammatory reactions by modulating inflammatory cascades which activate pro-inflammatory cytokines, such as TNF-α, thus attenuating chronic inflammation (Zugel and Kaufmann, 1999; Ikwegbue et al., 2019). In our study, HSP expression levels which were further decreased in the C and P groups, were upregulated in group D, thus alleviating diarrhea in neonatal calves. In agreement with the results of our study, Giri et al. (2018) and Kong et al. (2020) demonstrated that dietary supplementation with a probiotic mixture consisting of *B. subtilis*, *L. acidophilus*, and *S. cerevisiae* decreased the expression of HSP90 in in probiotic treated groups resulting in better growth and immunity.

Overexpression of HSP90S impairs the function of T cells, leading to a decrease in IL-2 expression (Bae et al., 2013). Enteric pathogens that invade a host are recognized by pattern recognition receptors (Kan et al., 2021). Probiotic supplementation can promote toll like receptors (TLR) to recognize various pathogens and activate NF-κB. Then NF-κB is transferred to the nucleus to induce the expression of target genes, thereby regulating immune and inflammatory responses (Huebener and Schwabe, 2013; Elghandour et al., 2020). This study shows that probiotic supplementation can effectively down regulate IL-1 in jejunal mucosa β, TNF-α, and NF-κ. The expression of B mRNA and inflammatory factors further indicates that probiotic supply can effectively resist *E. coli* infection by enhancing the intestinal innate immune function of newborn calves. This is consistent with the results of previous studies (Dong et al., 2019; Boranbayeva et al., 2020; Kan et al., 2021). However, some studies believe that the physiological functions of different probiotic strains are different (Kang and Im, 2015). Although the addition of probiotics can activate the TLR-NF-κB signaling pathway, the gene expression of IL-1β and TNF-α cytokines was not significantly affected (Wang et al., 2017). In conclusion, the gene expression of the TLR-NF-κB signaling pathway, pro-inflammatory cytokines, and heat shock protein in the jejunum of healthy newborn calves in group D in this article is down-regulated, indicating that antibiotic treatment of calves with diarrhea may reduce their immune levels and cause infections in the large intestine. The probiotic supplementation of bacillus new calves may focus on promoting the recovery of their intestinal function.

## Conclusion

We demonstrated that supplementation with MSP promotes immune function and microbiota function in neonatal dairy calves. MSP supplementation significantly changed the microbial structure and diversity of the small intestine of calves infected with *E. coli* K99 MSP supplementation improved intestinal immune function, especially the production of SIgA and SCFA concentrations in the jejunum of infected calves, decreased the relative mRNA expression of *IL-1*β and *TLR4* and increased the relative mRNA expression of *ZO-1* and *occludin* in the jejunal mucosa. Supplementation with MSP significantly enhanced intestinal energy metabolism and defense mechanisms of calves infected with *E. coli* K99 by reducing the expression of HSP90, which reduced inflammation and promoted the recovery of intestinal mucosal function. In view of these results, we recommend adding 2 g/day of MSP supplementation to the diets of dairy calves infected with *E. coli* K99. Our results are expected to provide a theoretical basis for the rational use of MSP supplements in calf production and provide substitutes for reducing the use of antibiotics.

## Data Availability Statement

The datasets presented in this study can be found in online repositories. The names of the repository/repositories and accession number(s) can be found in the article/Supplementary Material.

## Ethics Statement

This study was approved by the Ethics Committee of the College of Animal Science and Technology, Shihezi University (Shihezi, China) (No. A2020-171-01).

## Author Contributions

YW and WZ: conceptualization, writing—original draft preparation. YW, CN, RL, HC, JN, CC, and XB: investigation, writing—review and editing. YW and CN: formal analysis. All authors have read and approved the final version of the manuscript.

## Conflict of Interest

RL and HC were employed by Xinjiang Tianshan Junken Animal Husbandry Co., Ltd. The remaining authors declare that the research was conducted in the absence of any commercial or financial relationships that could be construed as a potential conflict of interest.

## Publisher’s Note

All claims expressed in this article are solely those of the authors and do not necessarily represent those of their affiliated organizations, or those of the publisher, the editors and the reviewers. Any product that may be evaluated in this article, or claim that may be made by its manufacturer, is not guaranteed or endorsed by the publisher.

## Footnotes

1
https://www.uniprot.org/

2
http://kobas.cbi.pku.edu.cn/
