# Supplementary material for: Effects of Multispecies Probiotic on Intestinal Microbiota and Mucosal Barrier Function of Neonatal Calves Infected With E. coli K99
Source: Front Microbiol. 2022 Jan 26;12:813245. doi: 10.3389/fmicb.2021.813245 (PMC8826468; doi:10.3389/fmicb.2021.813245)
Supplement: Supplementary file 1 [file Data_Sheet_1.doc]

**Effects of Multispecies Probioticon Intestinal Microbiota and Mucosal**

**Barrier Function of Neonatal Calves Infected With *E.coli* K99**

**Yanyan Wu**^1^, **Cunxi Nie**^1^**, Ruiqing Luo**^2^**, Hongli Chen**^2^**, Junli Niu**^1^**, Chen Chen^1^, Xue Bai^1^, Wenju Zhang***^1^

^1^College of Animal Science & Technology, Shihezi University, Shihezi, 832003, China;

^2^Xinjiang Tianshan Junken Animal Husbandry Co., Ltd. Shihezi , 832003, China

*^1^Corresponding author: Wenjun Zhang, College of Animal Science & Technology, Shihezi University, North 4th Road, Shihezi, 832003, China

1. mail: [zhangwj1022@sina.com)](mailto:zhangwj1022@sina.com))

Supplemental Table S1. Ingredient composition and nutrient levels of starter (DM basis)

| Item | Value |
| --- | --- |
| Ingredient, g / kg of DM |  |
| Corn | 55.20 |
| Soybean meal^1^ | 18.50 |
| Corn gluten meal | 10.00 |
| DGGS^2^ | 13.00 |
| Limestone | 1.80 |
| NaCl | 0.50 |
| Premix^3^ | 1.00 |
| Total | 100.00 |
| Chemical analysis |  |
| DM, g / kg | 87.33 |
| CP, g / kg | 19.92 |
| Ether extract, g / kg | 4.64 |
| ADF, g / kg | 6.02 |
| NDF, g / kg | 16.53 |
| Ash, g / kg | 5.38 |
| Calcium | 1.15 |
| Phosphorus | 0.58 |

^1^Soybean meal: 89.1% DM and 42.6% CP

^2^DDGS = distiller’s dried grains with solubles are the nutrient rich co-product of dry-milled ethanol production.

^3^ Premix provides the following per kg of the starter diet: VA 15,000 IU, VD 5000 IU, VE 50 mg, Fe 90 mg, Cu 12.5 mg, Mn 30 mg, Zn 90 mg, Se 0.3 mg, I 1.0 mg, Co 0.5 mg.

Supplemental Table S2. Primer sequences and annealing temperature

| Gene^1^ | Forward primer sequence 5’-3’  Reverse primer sequence 5’-3’ | Tm² (°C) | Fragment  length (bp) | Ta3 (°C) | Mean  quantification cycle (Cq) | Source | GenBank accession no. |
| --- | --- | --- | --- | --- | --- | --- | --- |
| Reference gene | | | | | | | |
| *β-actin* | GGGCGTAATGGTGGGCA | 60 | 217 | 60 |  | Veres-Székely et a3., 2017 | NM_ AB208021 |
|  | CTTGGGGTTCAGGGGGG | 60 |  |  |  |  |  |
| Genes of interest | | | | | | | |
| *TLR4* | GGGCAGGGAAAGTCAACTAAAC | 55 | 177 |  | 24.77 | Zhao, G et al., 2013 | NW_003104150 |
|  | TCAATCAAAACGACCAAAAACAC | 60 |  |  |  |  |  |
| *IL-1β* | AGGCTCTCCACCTCCTCTCA | 65 | 155 |  | 26.21 | Current paper | NM_214055.1 |
|  | GGGCGTATCACCTTTTTTCAC | 66 |  |  |  |  |  |
| *ZO-1* | GGAGAGGTGTTCCGTGTCG | 58 | 120 |  | 21.92 | Current paper | NM_008756.2 |
|  | TCTGTTTTTGTTTGGGATGATG | 59 |  |  |  |  |  |
| *Occludin* | ATCACTACACACCAAGCAATGAC | 60 | 194 |  | 23.26 | Current paper | NM_001163647.2 |
|  | GGCAACACAGGCGAAAAT | 60 |  |  |  |  |  |
| *Claudin* | TTCCTCTGCTTTTCCTGCTC | 60 | 161 |  | 24.88 | Current paper | NM_MF964215 |
|  | GTTCTTCACACATACCCCGTC | 60 |  |  |  |  |  |
| *TNF-α* | GCGGAGGTTCAGTGATGTAG | 54 | 224 |  | 24.91 | Current paper | NM_214022.1 |
|  | TTTATTCAGGAGGTCAAGGTGT | 68 |  |  |  |  |  |
| *NF-κB* | CGCAAAAGGACCTACGAGAC | 55 |  |  | 24.86 |  |  |
|  | TGGGGGAAAACTCATCAAAG | 60 |  |  |  |  |  |
| *IL-2* | CCTGAGCAGGATGGAGAATTACA | 65 | 141 |  | 24.43 | Mejret al(2011) |  |
|  | TCCAGAACATGCCGCAGAG | 66 |  |  |  |  |  |

# ^1^Toll-like receptor 4(*TLR4*); Interleukin-1 β(*IL-1β*);Zonula occludens 1(*ZO-1*);Tumor necrosis factor alpha (*TNF-α*);nuclear factor kappa-B(*NF-κB*); Interleukin-2 β(*IL-2*).

²Tm = melting temperature of the primer.

^3^Ta = annealing temperature, held for 30 s.

**References**

1.You, I., & Kim, E. B. Genome-based species-specific primers for rapid identification of six species of *Lactobacillus acidophilus* group using multiplex PCR. *PloS one*.  **2020,** 15(3), e0230550. <https://doi.org/10.1371/journal.pone.0230550.>

2.Chang, H. W., Nam, Y. D., Sung, Y., Kim, K. H., Roh, S. W., Yoon, J. H., An, K. G., & Bae, J. W. Quantitative real time PCR assays for the enumeration of *Saccharomyces cerevisiae* and the Saccharomyces sensu stricto complex in human feces. *Journal of microbiological methods*. **2007**.71(3), 191–201. https://doi.org/10.1016/j.mimet.2007.08.013.

3.Mejri N, Müller N, Hemphill A, Gottstein B ntraperitoneal *Echinococcus* multilocularis infection in mice modulates peritoneal CD4+ and CD8+ regulatory T cell development. *Parasitol Int*. **2011** Jan; 60(1):45-5.

4.Veres-Székely, Apor et al. “Selective measurement of α smooth muscle actin: why β-actin can not be used as a housekeeping gene when tissue fibrosis occurs.” *BMC molecular biology vol*. **2017**,18,1 12. 27 Apr. <https://doi.org/10.1186/s12867-017-0089-9.>

5. Zhao, G et al. “Association of bovine Toll-like receptor 4 with tick infestation rates and blood histamine concentration.” *Genetics and molecular research : GMR vol*. **2013**, 12,3 2783-93. 28 Feb. <https://doi.org/10.4238/2013.February.28.2>

Supplemental Table S3 The ASIOM analysis for digesta-associated microbial community

| Method name | R statistic | p-value | Number of permutations | Group |
| --- | --- | --- | --- | --- |
| Bacterial |  |  |  |  |
| Duodenum |  |  |  |  |
| ANOSIM | 0.64 | 0.006 | 999 | HD_D21-DD_D21 |
| ANOSIM | 0.48 | 0.009 | 999 | HD_D21-PD_D21 |
| ANOSIM | 0.93 | 0.002 | 999 | DD_D21-PD_D21 |
| ANOSIM | 0.68 | 0.001 | 999 | all |
| Jejunum |  |  |  |  |
| ANOSIM | 0.67 | 0.004 | 999 | HJ_D21-DJ_D21 |
| ANOSIM | 0.41 | 0.005 | 999 | HJ_D21-PJ_D21 |
| ANOSIM | 0.27 | 0.053 | 999 | DJ_D21-PJ_D21 |
| ANOSIM | 0.45 | 0.001 | 999 | all |
| Ileum |  |  |  |  |
| ANOSIM | 0.53 | 0.002 | 999 | HI_D21-DI_D21 |
| ANOSIM | 0.85 | 0.004 | 999 | HI_D21-PI_D21 |
| ANOSIM | 0.69 | 0.003 | 999 | DI_D21-PI_D21 |
| ANOSIM | 0.65 | 0.001 | 999 | all |
| Cecum |  |  |  |  |
| ANOSIM | 0.24 | 0.057 | 999 | HCa_D21-DCa_D21 |
| ANOSIM | 0.53 | 0.008 | 999 | HCa_D21-PCa_D21 |
| ANOSIM | 0.48 | 0.008 | 999 | DCa_D21-PCa_D21 |
| ANOSIM | 0.43 | 0.002 | 999 | all |
| Colon |  |  |  |  |
| ANOSIM | 0.42 | 0.007 | 999 | HCo_D21-DCo_D21 |
| ANOSIM | 0.72 | 0.002 | 999 | HCo_D21-PCo_D21 |
| ANOSIM | 0.39 | 0.025 | 999 | DCo_D21-PCo_D21 |
| ANOSIM | 0.51 | 0.001 | 999 | all |
| Rectum |  |  |  |  |
| ANOSIM | 0.79 | 0.002 | 999 | HRe_D21-DRe_D21 |
| ANOSIM | 0.55 | 0.011 | 999 | HRe_D21-PRe_D21 |
| ANOSIM | 0.57 | 0.006 | 999 | DRe_D21-PRe_D21 |
| ANOSIM | 0.63 | 0.001 | 999 | all |
| Fungus |  |  |  |  |
| Duodenum |  |  |  |  |
| ANOSIM | 0.06 | 0.188 | 999 | HD_D21-DD_D21 |
| ANOSIM | 0.27 | 0.013 | 999 | HD_D21-PD_D21 |
| ANOSIM | 0.26 | 0.016 | 999 | DD_D21-PD_D21 |
| ANOSIM | 0.19 | 0.02 | 999 | all |
| Jejunum |  |  |  |  |
| ANOSIM | 0.34 | 0.008 | 999 | HJ_D21-DJ_D21 |
| ANOSIM | 0.71 | 0.45 | 999 | HJ_D21-PJ_D21 |
| ANOSIM | 0.75 | 0.002 | 999 | DJ_D21-PJ_D21 |
| ANOSIM | 0.22 | 0.001 | 999 | all |
| Ileum |  |  |  |  |
| ANOSIM | 0.33 | 0.01 | 999 | HI_D21-DI_D21 |
| ANOSIM | 0.19 | 0.035 | 999 | HI_D21-PI_D21 |
| ANOSIM | 0.49 | 0.014 | 999 | DI_D21-PI_D21 |
| ANOSIM | 0.35 | 0.01 | 999 | all |
| Cecum |  |  |  |  |
| ANOSIM | 0.45 | 0.005 | 999 | HCa_D21-DCa_D21 |
| ANOSIM | 0.20 | 0.066 | 999 | HCa_D21-PCa_D21 |
| ANOSIM | 0.47 | 0.01 | 999 | DCa_D21-PCa_D21 |
| ANOSIM | 0.33 | 0.02 | 999 | all |
| Colon |  |  |  |  |
| ANOSIM | 0.16 | 0.042 | 999 | HCo_D21-DCo_D21 |
| ANOSIM | 0.13 | 0.157 | 999 | HCo_D21-PCo_D21 |
| ANOSIM | 0.26 | 0.046 | 999 | DCo_D21-PCo_D21 |
| ANOSIM | 0.18 | 0.06 | 999 | all |
| Rectum |  |  |  |  |
| ANOSIM | 0.56 | 0.005 | 999 | HRe_D21-DRe_D21 |
| ANOSIM | 0.09 | 0.191 | 999 | HRe_D21-PRe_D21 |
| ANOSIM | 0.24 | 0.044 | 999 | DRe_D21-PRe_D21 |
| ANOSIM | 0.32 | 0.06 | 999 | all |

Group (CD, Control Duodenum; DD, Diarrhea Duodenum; PD, MSP Duodenum; CJ, Control Jejunum; DJ, Diarrhea Jejunum; PJ, MSP Jejunum; CI, Control Ileum; DI, Diarrhea Ileum; PI, MSP Ileum; CCa, Control Cecum; DCa, Diarrhea Cecum; PCa, MSP Cecum; CCo, Control Colon; DCo, Diarrhea Colon; PCo, MSP Colon; CRe, Control Rectum; DRe, Diarrhea Rectum; PRe, MSP Rectum

Supplemental Table S4 The abundance and diversity of microbiota in neonatal calves fed with multispecies probiotics

| Item | Treatment (Trt)^1^ | | |  | *P*-value |
| --- | --- | --- | --- | --- | --- |
|  | C | D | P | SEM^2^ |  |
| Bacterial |  |  |  |  |  |
| Duodenum |  |  |  |  |  |
| Chao 1 | 215.58^b^ | 236.28^b^ | 381.01^a^ | 11.44 | <0.01 |
| Shannon | 3.9 | 4.61 | 4.99 | 0.04 | 0.24 |
| Simpson | 0.76 | 0.92 | 0.9 | 0.11 | 0.21 |
| Jejunum |  |  |  |  |  |
| Chao 1 | 324.15^ab^ | 190.11^b^ | 645.75^a^ | 46.74 | <0.01 |
| Shannon | 4.41^a^ | 2.52^b^ | 4.78^a^ | 0.02 | <0.01 |
| Simpson | 0.9^a^ | 0.65^b^ | 0.93^a^ | 0.01 | <0.01 |
| Ileum |  |  |  |  |  |
| Chao 1 | 218.26^b^ | 175.04^b^ | 348.09 | 15.24 | <0.01 |
| Shannon | 3.98^ab^ | 2.69^b^ | 4.94^a^ | 0.22 | 0 |
| Simpson | 0.87 | 0.68 | 0.92 | 0.02 | 0.01 |
| Cecum |  |  |  |  |  |
| Chao 1 | 323.32^b^ | 284.43^b^ | 572.05^a^ | 20.93 | 0.01 |
| Shannon | 4.66^a^ | 3.56^b^ | 4.87^a^ | 0.14 | 0.04 |
| Simpson | 0.93^ab^ | 0.78^b^ | 0.86^a^ | 0.01 | 0.06 |
| Colon |  |  |  |  |  |
| Chao 1 | 298.78 | 254.84 | 444.59 | 33.59 | 0.06 |
| Shannon | 4.52 | 3.36 | 4.56 | 0.17 | 0.08 |
| Simpson | 0.91 | 0.74 | 0.87 | 0.01 | 0.1 |
| Rectum |  |  |  |  |  |
| Chao 1 | 210.34^b^ | 246.18^b^ | 387.58^a^ | 7.44 | 0.02 |
| Shannon | 2.83^b^ | 4.23^a^ | 4.58^a^ | 0.25 | 0.001 |
| Simpson | 0.64^b^ | 0.89^a^ | 0.88^a^ | 0.06 | 0.001 |
| Fungal |  |  |  |  |  |
| Duodenum |  |  |  |  |  |
| Chao 1 | 30.57 | 34.12 | 47.55 | 4.39 | 0.26 |
| Shannon | 0.73 | 0.98 | 1.90 | 0.22 | 0.08 |
| Simpson | 0.19 | 0.25 | 0.55 | 0.06 | 0.046 |
| Jejunum |  |  |  |  |  |
| Chao 1 | 45.54 | 28.77 | 39.72 | 3.84 | 0.2 |
| Shannon | 2.70^a^ | 0.74b | 3.04^a^ | 0.32 | <0.01 |
| Simpson | 0.72^a^ | 0.17b | 0.77^a^ | 0.07 | <0.01 |
| Ileum |  |  |  |  |  |
| Chao 1 | 35.78 | 25.71 | 42.19 | 4.07 | 0.26 |
| Shannon | 0.48^b^ | 1.54^ab^ | 2.61^ab^ | 0.31 | 0.01 |
| Simpson | 0.14^b^ | 0.39^ab^ | 0.71^a^ | 0.08 | 0.01 |
| Cecum |  |  |  |  |  |
| Chao 1 | 75.05^a^ | 23.18^c^ | 50.03^b^ | 6.23 | <0.01 |
| Shannon | 1.54^a^ | 0.17^b^ | 1.87^a^ | 0.23 | <0.01 |
| Simpson | 0.40^a^ | 0.03^b^ | 0.49^a^ | 0.06 | <0.01 |
| Colon |  |  |  |  |  |
| Chao 1 | 63.58^a^ | 31.07^b^ | 48.37a^b^ | 5.11 | 0.02 |
| Shannon | 1.52 | 0.36 | 1.21 | 0.23 | 0.11 |
| Simpson | 0.41 | 0.1 | 0.32 | 0.06 | 0.16 |
| Rectum |  |  |  |  |  |
| Chao 1 | 93.64^a^ | 23.76c | 51.64b | 8.14 | <0.01 |
| Shannon | 1.97^a^ | 0.42^b^ | 1.16^a^ | 0.22 | <0.01 |
| Simpson | 0.57^a^ | 0.09^b^ | 0.33^a^ | 0.06 | <0.01 |

^a-b^ The values in the same row with different superscripts are significantly different (*P* < 0.05), while values with the same or no superscripts mean no significant difference (*P* ＞ 0.05).

^1^Treatments: 1)C ( Control group) treatment: fed a basal diet and not challenged with *E. coli* K99; 2) D (positive control, Diarrhea group): fed a basal diet and orally challenged with *E. coli* K99 (30 mL; 1×10^9^ CFU/mL) and Antibiotic support therapy（Intramuscular gentamicin 20 mL/ d）; 3) MSP treatment (MSP, Probiotics group): orally challenged with *E. coli* K99 (30 mL; 1.0×10^9^CFU/mL); fed a basal diet supplemented daily with MSP (7.0×10^9^CFU/g; 2 g/calf).

^2^SEM = Standard error of mean.

Supplemental Table S5 Taxonomic analysis of the principal intestinal segment phlya in neonatal calves, classified according to multispecies probiotics

| Phylum (%) | Treatment (Trt)1 | | |  |  |
| --- | --- | --- | --- | --- | --- |
|  | C | D | P | SEM^2^ | P |
| Bacteria |  |  |  |  |  |
| Duodenum |  |  |  |  |  |
| *p__Proteobacteria* | 3.01^c^ | 1.15^b^ | 13.52^a^ | 0.21 | 0.01 |
| *p__Firmicutes* | 32.44^b^ | 70.57^a^ | 33.23^b^ | 3.25 | 0.01 |
| *p__Actinobacteria* | 35.41^a^ | 6.01^b^ | 33.38^a^ | 3.90 | 0.01 |
| *p__Bacteroidetes* | 28.43 | 22.06 | 19.06 | 5.40 | 0.88 |
| Jejunum |  |  |  |  |  |
| *p__Proteobacteria* | 0.83^b^ | 0.67^b^ | 4.39^a^ | 0.21 | 0.03 |
| *p__Bacteroidetes* | 1.80^a^ | 0.07^b^ | 3.83^a^ | 0.25 | 0.03 |
| *p__Firmicutes* | 79.26^ab^ | 92.15^a^ | 71.19^b^ | 4.54 | 0.04 |
| *p__Actinobacteria* | 18.07^a^ | 6.97^b^ | 20.18^a^ | 0.59 | 0.03 |
| Ileum |  |  |  |  |  |
| *p__Firmicutes* | 86.42^ab^ | 57.61^c^ | 71.88^b^ | 4.65 | 0.01 |
| *p__Actinobacteria* | 7.04^b^ | 33.37^a^ | 5.99^b^ | 0.35 | 0.01 |
| *p__Fusobacteria* | 2.07^ac^ | 0.01^b^ | 0.00^c^ | 0.01 | 0.01 |
| *p__Cyanobacteria* | 0.06^a^ | 1.16^a^ | 0.00^b^ | 0.03 | 0.03 |
| *p__Proteobacteria* | 3.52^b^ | 3.69^a^ | 18.33^ab^ | 0.27 | 0.05 |
| *p__Bacteroidetes* | 0.59^b^ | 3.61^a^ | 2.95^b^ | 0.01 | 0.08 |
| Cecum |  |  |  |  |  |
| *p__Fusobacteria* | 2.59 | 0.02 | 0.75 | 0.01 | 0.37 |
| *p__Firmicutes* | 76.78 | 71.57 | 57.39 | 4.32 | 0.45 |
| *p__Proteobacteria* | 3.90 | 6.91 | 4.36 | 0.02 | 0.76 |
| *p__Actinobacteria* | 5.40 | 6.85 | 5.12 | 0.03 | 0.86 |
| *p__Bacteroidetes* | 11.17 | 14.56 | 32.35 | 1.21 | 0.86 |
| Colon |  |  |  |  |  |
| *p__Proteobacteria* | 5.59 | 0.22 | 17.69^a^ | 0.03 | 0.02 |
| *p__Fusobacteria* | 6.40^a^ | 0.01 | 3.87^a^ | 0.02 | 0.02 |
| *p__Firmicutes* | 50.78^b^ | 81.53^a^ | 59.63^b^ | 3.67 | 0.06 |
| *p__Actinobacteria* | 2.72 | 6.40 | 4.91 | 0.12 | 0.31 |
| *p__Bacteroidetes* | 34.47 | 11.42 | 13.78 | 0.23 | 0.85 |
| Rectum |  |  |  |  |  |
| *p__Fusobacteria* | 0.02^c^ | 0.42^b^ | 2.32^a^ | 0.01 | 0.01 |
| *p__Bacteroidetes* | 65.55^a^ | 23.92^b^ | 4.21^ab^ | 1.33 | 0.05 |
| *p__Firmicutes* | 26.52^b^ | 54.23^a^ | 64.54^a^ | 5.25 | 0.07 |
| *p__Verrucomicrobia* | 0.60 | 0.00 | 0.00 | 0.14 | 0.07 |
| *p__Proteobacteria* | 2.94c | 10.30^b^ | 18.90^ab^ | 0.26 | 0.07 |
| *p__Actinobacteria* | 4.36 | 11.06 | 10.02 | 0.11 | 0.10 |
| Fungi |  |  |  |  |  |
| Duodenum |  |  |  |  |  |
| *p__*Acaulium | 2.17^b^ | 24.67^a^ | 0.011^b^ | 3.89 | 0.01 |
| *p__*Aspergillus | 0.56^b^ | 3.58^a^ | 1.36^b^ | 0.43 | ＜0.01 |
| *p__*Filobasidium | 0.001^a^ | 0.001^a^ | 0.016^b^ | 0.00 | 0.01 |
| *p__*Kazachstania | 0.007^a^ | 0.001^a^ | 0.19^b^ | 0.03 | ＜0.01 |
| *p__*Saccharomyces | 88.04^a^ | 41.33^b^ | 84.40^a^ | 7.43 | ＜0.01 |
| *p__*Penicillium | 0.00^b^ | 0.00^b^ | 0.24^a^ | 0.03 | ＜0.01 |
| *p__*Acremonium | 0.00^b^ | 0.00^b^ | 0.33^a^ | 0.05 | ＜0.01 |
| *p__*Jejunum |  |  |  |  |  |
| *p__*Acaulium | 7.13^b^ | 20.88^a^ | 1.56^b^ | 2.64 | ＜0.01 |
| *p__*Cladosporium | 0.76^b^ | 2.97^a^ | 0.04^b^ | 0.39 | ＜0.01 |
| *p__*Kernia | 0.29^b^ | 0.82^a^ | 0.021^b^ | 0.12 | 0.01 |
| *p__*Pichia | 0.01^b^ | 2.37^a^ | 0.03^b^ | 0.37 | 0.01 |
| *p__*Saccharomyces | 28.13^b^ | 9.65^b^ | 89.17^a^ | 0.58 | ＜0.01 |
| *p__*Wardomyces | 3.87^b^ | 30.82^a^ | 0.06^b^ | 4.57 | ＜0.01 |
| *p__*Penicillium | 0.00 | 0.00 | 0.00 | 0.00 | NS |
| *p__*Acremonium | 0.00 | 0.00 | 0.00 | 0.00 | NS |
| *p__*Ileum |  |  |  |  |  |
| *p__*Acaulium | 0.12^b^ | 22.24^a^ | 0.04^b^ | 3.12 | ＜0.01 |
| *p__*Mortierella | 0.01^b^ | 10.63^a^ | 0.85^b^ | 2.04 | 0.05 |
| *p__*Saccharomyces | 81.49^a^ | 15.13^b^ | 61.28^a^ | 0.96 | 0.03 |
| *p__*Wardomyces | 0.00^b^ | 25.45^a^ | 0.00 ^b^ | 10.70 | 0.02 |
| Cecum |  |  |  |  |  |
| *p__*Acaulium | 3.57^b^ | 10.27^a^ | 0.004^b^ | 1.44 | 0.01 |
| *p__*Aspergillus | 4.34^a^ | 2.92^a^ | 0.19^b^ | 0.65 | 0.02 |
| *p__*Filobasidium | 0.017^a^ | 0.00^b^ | 0.00^b^ | 0.00 | 0.04 |
| *p__*Kernia | 0.23^a^ | 0.85^a^ | 0.00^b^ | 0.14 | 0.04 |
| *p__*Mortierella | 0.01^b^ | 4.80^a^ | 0.01^b^ | 0.87 | 0.02 |
| *p__*Saccharomyces | 67.45^b^ | 42.06^b^ | 98.10a | 8.61 | 0.01 |
| *p__*Wardomyces | 0.018^b^ | 29.66^a^ | 0.01^b^ | 5.61 | 0.03 |
| *p__*Colon |  |  |  |  |  |
| *p__*Saccharomyces | 66.82^b^ | 48.84^b^ | 94.15^a^ | 9.23 | 0.02 |
| *p__*Scopulariopsis | 0.21^b^ | 1.22^a^ | 0.05^b^ | 0.18 | 0.01 |
| *p__*Thielavia | 0.02 | 0.00 | 0.00 | 0.00 | 0.28 |
| *p__*Wardomyces | 0.015^b^ | 32.11^a^ | 0.025^b^ | 5.81 | 0.02 |
| Rectum |  |  |  |  |  |
| *p__*Wardomyces | 0.10^b^ | 29.31^a^ | 0.003^b^ | 5.29 | 0.02 |

^a-b^ The values in the same row with different superscripts are significantly different (*P* < 0.05), while values with the same or no superscripts mean no significant difference (*P* ＞ 0.05).

^1^Treatments: 1)C ( Control group) treatment: fed a basal diet and not challenged with *E. coli* K99; 2) D (positive control, Diarrhea group): fed a basal diet and orally challenged with *E. coli* K99 (30 mL; 1×10^9^ CFU/mL) and Antibiotic support therapy（Intramuscular gentamicin 20 mL/ d）; 3) MSP treatment (MSP, Probiotics group): orally challenged with *E. coli* K99 (30 mL; 1.0×10^9^ CFU/mL); fed a basal diet supplemented daily with MSP (7.0×10^9^CFU/g; 2 g/calf).

^2^SEM = Standard error of mean.

Supplemental Table S6 Taxonomic analysis of the principal intestinal segment genus in in neonatal calves classified according to treatment

| Genus (%) | Treatment (Trt)1 | | |  |  |
| --- | --- | --- | --- | --- | --- |
|  | C | D | P | SEM2 | P |
| Bacterial |  |  |  |  |  |
| Duodenum |  |  |  |  |  |
| g__Bacteroides | 0.03^b^ | 0.09^b^ | 0.90^a^ | 0.14 | 0.02 |
| g__Lactobacillus | 1.04^b^ | 12.04^a^ | 3.04^b^ | 0.31 | <0.01 |
| g__Olsenella | 31.26 | 5.08 | 9.1 | 5.3 | 0.08 |
| g__Sarcina | 0.00^c^ | 8.55^a^ | 0.52^b^ | 0.95 | <0.01 |
| g__Blautia | 0.65^a^ | 0.01^b^ | 0.51^a^ | 0.1 | 0.02 |
| g__Escherichia−Shigella | 0.37 | 0.01 | 0.07 | 0.08 | 0.14 |
| g__Bifidobacterium | 2.16^b^ | 0.40^c^ | 17.83^a^ | 0.02 | 0.02 |
| g__Sharpea | 4.55^ab^ | 0.71^b^ | 6.94^a^ | 0.01 | 0.09 |
| g__Faecalibacterium | 0.70^a^ | 0.00^b^ | 0.03^b^ | 0.12 | 0.02 |
| g__Streptococcus | 0.45^b^ | 4.32^a^ | 0.12^c^ | 0.46 | <0.01 |
| g__Ruminococcaceae_UCG−005 | 0 | 0 | 0.02 | 0.01 | 0.14 |
| g__Syntrophococcus | 2.45^a^ | 0.40^b^ | 0.90^b^ | 0.32 | 0.01 |
| g__Prevotella_7 | 17.99^a^ | 8.97^ab^ | 2.16^b^ | 2.42 | 0.02 |
| g__Selenomonas | 3.19^b^ | 20.17^a^ | 0.16^c^ | 2.12 | <0.01 |
| g__Tyzzerella_4 | 0.02^a^ | 0.00^b^ | 0.00^b^ | 0.003 | 0.04 |
| g__Enterococcus | 0.91^a^ | 0.12^b^ | 0.01^b^ | 0.1 | <0.01 |
| g__Collinsella | 0.73^a^ | 0.01^b^ | 0.06^b^ | 0.12 | 0.03 |
| g__Ruminococcaceae_UCG−014 | 1.59 | 0.04 | 2.62 | 0.52 | 0.12 |
| g__Romboutsia | 0.04 | 0.03 | 0.06 | 0.01 | 0.08 |
| g__Eubacterium_coprostanoligenes_group | 0.03^b^ | 0.15^b^ | 0.78^a^ | 0.11 | 0.01 |
| **Jejunum** |  |  |  |  |  |
| g__Bacteroides | 0.06^a^ | 0.01^b^ | 0.11^ab^ | 0.01 | 0.04 |
| g__Lactobacillus | 1.08 | 17.02 | 18.74 | 5.23 | 0.61 |
| g__Olsenella | 13.15 | 6.47 | 14.23 | 2.47 | 0.4 |
| g__Sarcina | 0.00^b^ | 26.66^a^ | 3.63^b^ | 5.02 | 0.05 |
| g__Blautia | 0.38^a^ | 0.03^b^ | 0.03^b^ | 0.06 | 0.03 |
| g__Escherichia−Shigella | 0.03^a^ | 0.45^b^ | 0.02^a^ | 0.08 | 0.04 |
| g__Bifidobacterium | 2.04^a^ | 0.14^b^ | 4.21^a^ | 0.62 | 0.02 |
| g__Sharpea | 10.12^a^ | 0.34^b^ | 5.33^a^ | 1.47 | 0.02 |
| g__Faecalibacterium | 0.03^a^ | 0.00^b^ | 0.01^b^ | 0 | <0.01 |
| g__Streptococcus | 1.9 | 0.54 | 0.05 | 0.4 | 0.15 |
| g__Ruminococcaceae_UCG−005 | 0.02^a^ | 0.00^b^ | 0.01a^b^ | 0 | 0.02 |
| g__Syntrophococcus | 20.21^a^ | 2.13^b^ | 2.56^b^ | 2.66 | <0.01 |
| g__Prevotella_7 | 1.10^ab^ | 0.02^b^ | 2.98^a^ | 0.5 | 0.04 |
| g__Selenomonas | 0.1 | 0.19 | 0.16 | 0.04 | 0.42 |
| g__Tyzzerella_4 | 0 | 0 | 0 | 0 | 0.29 |
| g__Enterococcus | 1.54 | 8.37 | 0.01 | 1.68 | 0.08 |
| g__Collinsella | 0.03 | 0.05 | 0.02 | 0.01 | 0.51 |
| g__Ruminococcaceae_UCG−014 | 2.66^a^ | 0.07^b^ | 2.13^a^ | 0.38 | 0.01 |
| g__Romboutsia | 0.02^b^ | 20.21^a^ | 0.17^b^ | 3.66 | 0.02 |
| g__Eubacterium_coprostanoligenes_group | 0.03 | 0 | 0 | 0 | 0.02 |
| Ileum |  |  |  |  |  |
| g__Bacteroides | 0.12 | 0.37 | 0.36 | 0.06 | 0.17 |
| g__Lactobacillus | 41.45^a^ | 15.20^ab^ | 2.21^b^ | 6.88 | 0.05 |
| g__Olsenella | 4.2 | 6.37 | 8.05 | 1.25 | 0.48 |
| g__Sarcina | 0.00^b^ | 33.77^a^ | 0.12^b^ | 5.78 | 0.01 |
| g__Blautia | 0.07^b^ | 0.03^b^ | 0.35^a^ | 0.05 | <0.01 |
| g__Escherichia−Shigella | 8.83 | 3.18 | 0.05 | 1.61 | 0.07 |
| g__Bifidobacterium | 0.66^b^ | 0.08^b^ | 20.32^a^ | 2.5 | 0.01 |
| g__Sharpea | 3.73^b^ | 0.13^b^ | 13.99^a^ | 1.64 | <0.01 |
| g__Faecalibacterium | 0.05 | 0.07 | 0.05 | 0.01 | 0.72 |
| g__Streptococcus | 5.21^a^ | 0.28 ^b^ | 0.56^b^ | 0.75 | <0.01 |
| g__Ruminococcaceae_UCG−005 | 0.00^b^ | 0.03^b^ | 0.77^a^ | 0.1 | 0.01 |
| g__Syntrophococcus | 0.54 | 7.64 | 2.25 | 1.37 | 0.08 |
| g__Prevotella_7 | 1.27 | 0.03 | 0.12 | 0.27 | 0.12 |
| g__Selenomonas | 4.01 | 0.08 | 4.59 | 1.13 | 0.22 |
| g__Tyzzerella_4 | 0.00^b^ | 0.06^a^ | 0.05^a^ | 0.01 | <0.01 |
| g__Enterococcus | 3.23 | 9.18 | 0.03 | 1.71 | 0.08 |
| g__Collinsella | 0.01^b^ | 0.08^b^ | 0.28^a^ | 0.04 | 0.01 |
| g__Ruminococcaceae_UCG−014 | 0.12^b^ | 0.21^b^ | 2.16^a^ | 0.34 | 0.01 |
| g__Romboutsia | 0.24^b^ | 0.56^b^ | 1.14^a^ | 0.13 | 0.01 |
| g__Eubacterium_coprostanoligenes_group | 0.00^b^ | 0.78^ab^ | 2.24^a^ | 0.36 | 0.03 |
| **Cecum** |  |  |  |  |  |
| g__Bacteroides | 11.68 | 31.98 | 3.22 | 5.6 | 0.1 |
| g__Lactobacillus | 10.86 | 6.56 | 9.42 | 2.95 | 0.85 |
| g__Olsenella | 1.67 | 0.44 | 0.93 | 0.24 | 0.12 |
| g__Sarcina | 0.00^b^ | 2.85^a^ | 0.12^b^ | 0.56 | 0.05 |
| g__Blautia | 12.57^a^ | 3.13^b^ | 8.10^ab^ | 1.43 | 0.02 |
| g__Escherichia−Shigella | 4.6 | 4.26 | 3.38 | 1.39 | 0.94 |
| g__Bifidobacterium | 1.14 | 1.3 | 2.69 | 0.36 | 0.17 |
| g__Sharpea | 1.88 | 0.47 | 0.69 | 0.37 | 0.52 |
| g__Faecalibacterium | 4.60^ab^ | 7.43^a^ | 0.28^b^ | 1.15 | 0.03 |
| g__Streptococcus | 6.13 | 5.79 | 0.08 | 1.28 | 0.1 |
| g__Ruminococcaceae_UCG−005 | 0.00^b^ | 0.71^b^ | 22.27^a^ | 3.51 | <0.01 |
| g__Syntrophococcus | 0.13 | 0.02 | 0.14 | 0.03 | 0.2 |
| g__Prevotella_7 | 0.01 | 0.05 | 0.43 | 0.13 | 0.35 |
| g__Selenomonas | 0.01 | 0.02 | 0.02 | 0.01 | 0.78 |
| g__Tyzzerella_4 | 8.72 | 7.89 | 0.31 | 2.21 | 0.24 |
| g__Enterococcus | 0.42 | 0.68 | 0.04 | 0.16 | 0.29 |
| g__Collinsella | 3.52 | 3.28 | 1.49 | 0.71 | 0.47 |
| g__Ruminococcaceae_UCG−014 | 1.35^b^ | 0.02^b^ | 3.92^a^ | 0.57 | <0.01 |
| g__Romboutsia | 0.35 | 0.31 | 0.03 | 0.09 | 0.31 |
| g__Eubacterium_coprostanoligenes_group | 0.00^b^ | 0.03^b^ | 3.46^a^ | 0.53 | <0.01 |
| **Colon** |  |  |  |  |  |
| g__Bacteroides | 8.11 | 34.47 | 5.05 | 5.87 | 0.07 |
| g__Lactobacillus | 4.95 | 6.29 | 2.87 | 1.56 | 0.69 |
| g__Olsenella | 2.12^a^ | 0.35^b^ | 0.39^b^ | 0.33 | 0.03 |
| g__Sarcina | 0.00^b^ | 4.60^a^ | 0.35^b^ | 0.81 | 0.03 |
| g__Blautia | 19.01^a^ | 2.47^b^ | 6.69^b^ | 2.09 | <0.01 |
| g__Escherichia−Shigella | 0.15 | 4.94 | 17.08 | 3.36 | 0.16 |
| g__Bifidobacterium | 1.99 | 0.84 | 1.94 | 0.47 | 0.42 |
| g__Sharpea | 2.9 | 0.11 | 0.48 | 0.56 | 0.08 |
| g__Faecalibacterium | 13.21^a^ | 4.16^b^ | 0.40 ^b^ | 1.64 | <0.01 |
| g__Streptococcus | 2.86 | 4.38 | 0.04 | 0.81 | 0.08 |
| g__Ruminococcaceae_UCG−005 | 0.00^b^ | 0.59^b^ | 15.23^a^ | 2.43 | <0.01 |
| g__Syntrophococcus | 0.16 | 0.02 | 0.03 | 0.03 | 0.1 |
| g__Prevotella_7 | 0.04 | 0.02 | 0.15 | 0.03 | 0.3 |
| g__Selenomonas | 0.02 | 0.02 | 0.01 | 0.01 | 0.75 |
| g__Tyzzerella_4 | 3.47 | 4.82 | 0.26 | 0.95 | 0.13 |
| g__Enterococcus | 0.28 | 3.33 | 0.07 | 0.94 | 0.3 |
| g__Collinsella | 1.54 | 1.42 | 2.38 | 0.43 | 0.92 |
| g__Ruminococcaceae_UCG−014 | 2.27 | 0.01 | 3.43 | 1.29 | 0.07 |
| g__Romboutsia | 0.01 | 0.47 | 0.02 | 0.1 | 0.1 |
| g__Eubacterium_coprostanoligenes_group | 0.00^b^ | 0.04^b^ | 4.46^a^ | 0.63 | <0.01 |
| **Rectum** |  |  |  |  |  |
| g__Bacteroides | 8.11^b^ | 34.47^a^ | 5.05^b^ | 5.87 | 0.07 |
| g__Lactobacillus | 4.95 | 6.29 | 2.87 | 1.56 | 0.69 |
| g__Olsenella | 2.12^a^ | 0.35^b^ | 0.39^b^ | 0.33 | 0.03 |
| g__Sarcina | 0 | 0.06^a^ | 0.05^b^ | 0.01 | 0.04 |
| g__Blautia | 19.01^a^ | 2.47^b^ | 6.69^b^ | 2.09 | <0.01 |
| g__Escherichia−Shigella | 0.15 | 4.94 | 17.08 | 3.35 | 0.1 |
| g__Bifidobacterium | 1.99 | 0.84 | 1.94 | 0.41 | 0.47 |
| g__Sharpea | 2.9 | 0.11 | 0.48 | 0.56 | 0.08 |
| g__Faecalibacterium | 13.21 | 4.16 | 0.4 | 1.64 | <0.01 |
| g__Streptococcus | 2.86 | 4.38 | 0.04 | 0.8 | 0.08 |
| g__Ruminococcaceae_UCG−005 | 0 | 0.59 | 15.23 | 2.43 | <0.01 |
| g__Syntrophococcus | 0.16 | 0.02 | 0.03 | 0.03 | 0.19 |
| g__Prevotella_7 | 0.04 | 0.02 | 0.15 | 0.03 | 0.3 |
| g__Selenomonas | 0.02 | 0.02 | 0.01 | 0 | 0.69 |
| g__Tyzzerella_4 | 3.47 | 4.82 | 0.26 | 0.95 | 0.13 |
| g__Enterococcus | 0.28 | 3.33 | 0.07 | 0.95 | 0.3 |
| g__Collinsella | 1.54 | 1.42 | 2.38 | 0.43 | 0.64 |
| g__Ruminococcaceae_UCG−014 | 2.27^a^ | 0.01^b^ | 3.43^a^ | 0.62 | 0.07 |
| g__Romboutsia | 0.01 | 0.47 | 0.02 | 0.1 | 0.1 |
| g__Eubacterium_coprostanoligenes_group | 0.00^b^ | 0.04^b^ | 4.46^a^ | 0.64 | <0.01 |
| Fungi |  |  |  |  |  |
| Duodenum |  |  |  |  |  |
| *g__Acaulium* | 2.17b | 24.67a | 0.011b | 3.89 | 0.01 |
| *g__Acrostalagmus* | 0.02 | 0.33 | 0.00 | 0.07 | 0.13 |
| *g__Alternaria* | 0.01 | 0.02 | 0.05 | 0.01 | 0.17 |
| *g__Apiotrichum* | 0.12 | 1.53 | 1.83 | 0.57 | 0.45 |
| *g__Aspergillus* | 0.56b | 3.58a | 1.36b | 0.43 | ＜0.01 |
| *g__Cladosporium* | 0.00 | 0.67 | 0.01 | 0.21 | 0.37 |
| *g__Cutaneotrichosporon* | 0.31 | 0.19 | 0.42 | 0.08 | 0.60 |
| *g__Filobasidium* | 0.001a | 0.001a | 0.016b | 0.00 | 0.01 |
| *g__Geotrichum* | 1.19 | 0.40 | 6.50 | 1.21 | 0.08 |
| *g__Kazachstania* | 0.007a | 0.001a | 0.19b | 0.03 | ＜0.01 |
| *g__Kernia* | 0.09 | 0.65 | 0.78 | 0.18 | 0.28 |
| *g__Mortierella* | 2.67 | 2.11 | 0.01 | 1.09 | 0.61 |
| *g__Naganishia* | 0.00 | 0.10 | 1.49 | 0.34 | 0.13 |
| *g__Pichia* | 0.00 | 0.68 | 0.00 | 0.22 | 0.39 |
| *g__Saccharomyces* | 88.04a | 41.33b | 84.40a | 7.43 | ＜0.01 |
| *g__Scopulariopsis* | 0.23 | 2.41 | 0.00 | 0.62 | 0.23 |
| *g__Thielavia* | 0.00 | 0.00 | 0.00 | 0.00 | NS |
| *g__Wardomyces* | 1.26 | 17.93 | 0.00 | 4.08 | 0.13 |
| *g__Penicillium* | 0.00b | 0.00b | 0.24a | 0.03 | ＜0.01 |
| *g__Acremonium* | 0.00b | 0.00b | 0.33a | 0.05 | ＜0.01 |
| Jejunum |  |  |  |  |  |
| *g__Acaulium* | 7.13b | 20.88a | 1.56b | 2.64 | ＜0.01 |
| *g__Acrostalagmus* | 0.09 | 0.00 | 0.00 | 0.03 | 0.36 |
| *g__Alternaria* | 11.59 | 0.88 | 0.00 | 2.65 | 0.13 |
| *g__Apiotrichum* | 0.43 | 8.99 | 0.02 | 1.98 | 0.11 |
| *g__Aspergillus* | 3.25 | 7.71 | 2.81 | 1.57 | 0.39 |
| *g__Cladosporium* | 0.76b | 2.97a | 0.04b | 0.39 | ＜0.01 |
| *g__Cutaneotrichosporon* | 2.37 | 2.94 | 0.15 | 0.92 | 0.45 |
| *g__Filobasidium* | 5.35 | 0.15 | 0.01 | 1.22 | 0.13 |
| *g__Geotrichum* | 12.55 | 0.88 | 1.64 | 3.95 | 0.42 |
| *g__Kazachstania* | 0.01 | 0.07 | 0.20 | 0.04 | 0.13 |
| *g__Kernia* | 0.29^b^ | 0.82^a^ | 0.021^b^ | 0.12 | 0.01 |
| *g__Mortierella* | 7.71 | 1.61 | 0.05 | 1.43 | 0.06 |
| *g__Naganishia* | 0.00 | 0.00 | 0.79 | 0.25 | 0.37 |
| *g__Pichia* | 0.01^b^ | 2.37^a^ | 0.03^b^ | 0.37 | 0.01 |
| *g__Saccharomyces* | 28.13^b^ | 9.65^b^ | 89.17^a^ | 0.58 | ＜0.01 |
| *g__Scopulariopsis* | 0.68 | 1.30 | 0.05 | 0.26 | 0.14 |
| *g__Thielavia* | 0.00 | 0.00 | 0.00 | 0.00 | NS |
| *g__Wardomyces* | 3.87^b^ | 30.82^a^ | 0.06^b^ | 4.57 | ＜0.01 |
| *g__Penicillium* | 0.00 | 0.00 | 0.00 | 0.00 | NS |
| *g__Acremonium* | 0.00 | 0.00 | 0.00 | 0.00 | NS |
| Ileum |  |  |  |  |  |
| *g__Acaulium* | 0.12^b^ | 22.24^a^ | 0.04^b^ | 3.12 | ＜0.01 |
| *g__Acrostalagmus* | 0.05 | 0.18 | 0.00 | 0.05 | 0.37 |
| *g__Alternaria* | 0.04 | 0.00 | 0.22 | 0.06 | 0.40 |
| *g__Apiotrichum* | 0.01 | 0.85 | 4.63 | 1.04 | 0.15 |
| *g__Aspergillus* | 1.79 | 9.49 | 3.58 | 2.01 | 0.27 |
| *g__Cladosporium* | 0.01 | 0.74 | 0.00 | 0.17 | 0.13 |
| *g__Cutaneotrichosporon* | 0.70 | 1.47 | 0.78 | 0.40 | 0.71 |
| *g__Filobasidium* | 0.02 | 0.00 | 0.00 | 0.01 | 0.39 |
| *g__Geotrichum* | 15.27 | 0.04 | 13.25 | 5.29 | 0.47 |
| *g__Kazachstania* | 0.00 | 0.00 | 1.99 | 0.63 | 0.36 |
| *g__Kernia* | 0.00 | 0.90 | 2.15 | 0.47 | 0.18 |
| *g__Mortierella* | 0.01^b^ | 10.63^a^ | 0.85^b^ | 2.04 | 0.05 |
| *g__Naganishia* | 0.00 | 0.03 | 4.16 | 0.89 | 0.08 |
| *g__Pichia* | 0.01 | 0.10 | 0.02 | 0.03 | 0.52 |
| *g__Saccharomyces* | 81.49^a^ | 15.13^b^ | 61.28^a^ | 0.96 | 0.03 |
| *g__Scopulariopsis* | 0.00 | 6.82 | 0.27 | 1.47 | 0.09 |
| *g__Thielavia* | 0.00 | 0.00 | 0.00 | 0.00 | NS |
| *g__Wardomyces* | 0.00^b^ | 25.45^a^ | 0.00^b^ | 10.70 | 0.02 |
| *g__Penicillium* | 0.00 | 0.00 | 0.00 | 0.00 | NS |
| *g__Acremonium* | 0.00 | 0.00 | 0.00 | 0.00 | NS |
| Cecum |  |  |  |  |  |
| *g__Acaulium* | 3.57^b^ | 10.27^a^ | 0.004^b^ | 1.44 | 0.01 |
| *g__Acrostalagmus* | 0.74^a^ | 0.17^ab^ | 0.00^b^ | 0.13 | 0.07 |
| *g__Alternaria* | 0.26 | 0.01 | 0.00 | 0.06 | 0.12 |
| *g__Apiotrichum* | 0.07 | 0.76 | 0.06 | 0.24 | 0.42 |
| *g__Aspergillus* | 4.34^a^ | 2.92^a^ | 0.19^b^ | 0.65 | 0.02 |
| *g__Cladosporium* | 0.05 | 0.29 | 0.00 | 0.08 | 0.38 |
| *g__Cutaneotrichosporon* | 9.78 | 1.10 | 0.02 | 2.62 | 0.26 |
| *g__Filobasidium* | 0.017^a^ | 0.00^b^ | 0.00^b^ | 0.00 | 0.04 |
| *g__Geotrichum* | 10.09 | 0.05 | 0.62 | 2.05 | 0.07 |
| *g__Kazachstania* | 0.05 | 0.00 | 0.43 | 0.09 | 0.10 |
| *g__Kernia* | 0.23^a^ | 0.85^a^ | 0^b^ | 0.14 | 0.04 |
| *g__Mortierella* | 0.01^b^ | 4.80^a^ | 0.001^b^ | 0.87 | 0.02 |
| *g__Naganishia* | 0.01 | 0.12 | 0.00 | 0.03 | 0.19 |
| *g__Pichia* | 0.06 | 0.10 | 0.01 | 0.03 | 0.59 |
| *g__Saccharomyces* | 67.45^b^ | 42.06^b^ | 98.10^a^ | 8.61 | 0.01 |
| *g__Scopulariopsis* | 0.19 | 2.98 | 0.01 | 0.62 | 0.08 |
| *g__Thielavia* | 0.00 | 0.00 | 0.00 | 0.00 | NS |
| *g__Wardomyces* | 0.02^b^ | 29.66^a^ | 0.01^b^ | 5.61 | 0.03 |
| *g__Penicillium* | 0.00 | 0.00 | 0.30 | 0.06 | 0.10 |
| *g__Acremonium* | 0.00 | 0.00 | 0.00 | 0.00 | NS |
| Colon |  |  |  |  |  |
| *g__Acaulium* | 4.66 | 8.51 | 0.26 | 1.77 | 0.16 |
| *g__Acrostalagmus* | 1.30 | 0.01 | 0.00 | 0.37 | 0.28 |
| *g__Alternaria* | 0.06 | 0.03 | 3.17 | 1.04 | 0.39 |
| *g__Apiotrichum* | 0.22 | 0.16 | 0.04 | 0.05 | 0.38 |
| *g__Aspergillus* | 13.09^a^ | 0.93^ab^ | 0.11^b^ | 2.64 | 0.07 |
| *g__Cladosporium* | 0.06 | 0.10 | 0.02 | 0.02 | 0.35 |
| *g__Cutaneotrichosporon* | 2.61 | 0.07 | 0.08 | 0.53 | 0.07 |
| *g__Filobasidium* | 0.04 | 0.00 | 0.00 | 0.01 | 0.15 |
| *g__Geotrichum* | 8.43 | 0.02 | 0.99 | 1.84 | 0.12 |
| *g__Kazachstania* | 0.01 | 0.03 | 0.10 | 0.02 | 0.16 |
| *g__Kernia* | 0.45 | 1.04 | 0.00 | 0.21 | 0.13 |
| *g__Mortierella* | 0.03^b^ | 4.56^a^ | 0.00^b^ | 0.83 | 0.03 |
| *g__Naganishia* | 0.00 | 0.01 | 0.03 | 0.01 | 0.33 |
| *g__Pichia* | 0.00 | 0.00 | 0.00 | 0.00 | NS |
| *g__Saccharomyces* | 66.82^b^ | 48.84^b^ | 94.15^a^ | 9.23 | 0.02 |
| *g__Scopulariopsis* | 0.21^b^ | 1.22^a^ | 0.05^b^ | 0.18 | 0.01 |
| *g__Thielavia* | 0.02 | 0.00 | 0.00 | 0.00 | 0.28 |
| *g__Wardomyces* | 0.015^b^ | 32.11^a^ | 0.025^b^ | 5.81 | 0.02 |
| *g__Penicillium* | 0.00 | 0.00 | 0.08 | 0.01 | 0.10 |
| *g__Acremonium* | 0.00 | 0.00 | 0.24 | 0.04 | 0.00 |
| Rectum |  |  |  |  |  |
| *g__Acaulium* | 24.40^a^ | 9.25^b^ | 0.69^b^ | 3.60 | 0.01 |
| *g__Acrostalagmus* | 4.02 | 0.05 | 0.00 | 1.22 | 0.32 |
| *g__Alternaria* | 0.03 | 0.00 | 0.03 | 0.01 | 0.36 |
| *g__Apiotrichum* | 0.09 | 0.01 | 0.20 | 0.04 | 0.23 |
| *g__Aspergillus* | 11.83 | 0.88 | 1.00 | 2.44 | 0.11 |
| *g__Cladosporium* | 0.027^a^ | 0.002^b^ | 0.004^b^ | 0.00 | 0.00 |
| *g__Cutaneotrichosporon* | 0.89^a^ | 0.006^b^ | 0.027^b^ | 0.13 | 0.00 |
| *g__Filobasidium* | 0.00 | 0.00 | 0.00 | 0.00 | NS |
| *g__Geotrichum* | 4.18 | 0.03 | 0.82 | 1.15 | 0.31 |
| *g__Kazachstania* | 0.00^b^ | 0.00^b^ | 0.27^a^ | 0.05 | 0.03 |
| *g__Kernia* | 1.01^a^ | 0.64^a^ | 0.001^b^ | 0.16 | 0.02 |
| *g__Mortierella* | 0.01 | 8.13 | 0.00 | 1.73 | 0.08 |
| *g__Naganishia* | 0.01^a^ | 0.003^a^ | 0.00^b^ | 0.00 | 0.06 |
| *g__Pichia* | 0.00 | 0.00 | 0.01 | 0.00 | 0.53 |
| *g__Saccharomyces* | 50.38^b^ | 49.08^b^ | 93.22 | 9.20 | 0.08 |
| *g__Scopulariopsis* | 0.61 | 0.99 | 0.02 | 0.23 | 0.24 |
| *g__Thielavia* | 0.00 | 0.00 | 1.67 | 0.51 | 0.33 |
| *g__Wardomyces* | 0.10^b^ | 29.31^a^ | 0.003^b^ | 5.29 | 0.02 |

Supplemental Table S7 Gene ontology enrichment analysis shows the host proteins demonstrated that the mostly enriched biological functions

|  | Treatment (Trt)^1^ | |  |  |  |
| --- | --- | --- | --- | --- | --- |
| Protein | P | D | log2FC | score | FDR |
| A0A140T866 | 44110969.83 | 2970649.60 | 3.89 | 0.98 | 0.01 |
| A0A140T872 | 1049643771.87 | 13634121780.57 | -3.70 | 0.94 | 0.02 |
| A0A140T876 | 512567764.49 | 29652932.87 | 4.11 | 0.95 | 0.02 |
| A0A2D3UL72 | 71392965.28 | 4329119.38 | 4.04 | 0.94 | 0.02 |
| A0A3Q1LM20 | 25275319666.55 | 946836624.76 | 4.74 | 0.99 | 0.00 |
| A0A3Q1LMQ9 | 2371569375.01 | 156729608.91 | 3.92 | 0.91 | 0.03 |
| A0A3Q1LMV5 | 2919446904.77 | 259105596.98 | 3.49 | 0.98 | 0.01 |
| A0A3Q1LPH5 | 309232060.98 | 13400366.54 | 4.53 | 0.96 | 0.02 |
| A0A3Q1LR19 | 365059425.90 | 8878525.01 | 5.36 | 0.97 | 0.01 |
| A0A3Q1LR42 | 1313544809.85 | 144359505.91 | 3.19 | 0.91 | 0.03 |
| A0A3Q1LR67 | 920834827.47 | 33589906.88 | 4.78 | 0.99 | 0.00 |
| A0A3Q1LT07 | 75751249.01 | 5922856.86 | 3.68 | 0.98 | 0.01 |
| A0A3Q1LVI9 | 11302635.41 | 1167784.67 | 3.27 | 0.97 | 0.01 |
| A0A3Q1LZQ5 | 1482100030.68 | 81344118.79 | 4.19 | 0.95 | 0.02 |
| A0A3Q1LZY6 | 110592836.44 | 3006082.84 | 5.20 | 1.00 | 0.00 |
| A0A3Q1M2K6 | 1182887890.42 | 76453022.99 | 3.95 | 0.98 | 0.01 |
| A0A3Q1M315 | 59424454.97 | 4946095.97 | 3.59 | 0.95 | 0.02 |
| A0A3Q1M3A8 | 147878970.86 | 16016801.72 | 3.21 | 0.93 | 0.02 |
| A0A3Q1M3W5 | 10778227018.37 | 40199266.69 | 8.07 | 0.98 | 0.01 |
| A0A3Q1M5N0 | 501200524.94 | 26432506.48 | 4.25 | 0.87 | 0.05 |
| A0A3Q1M6R5 | 1823555832.32 | 83821248.88 | 4.44 | 1.00 | 0.00 |
| A0A3Q1MB49 | 1719269389.83 | 110788552.04 | 3.96 | 0.98 | 0.01 |
| A0A3Q1MBF5 | 2400062446.63 | 147688375.43 | 4.02 | 0.93 | 0.03 |
| A0A3Q1MDB8 | 6947791733.60 | 402722177.87 | 4.11 | 0.97 | 0.01 |
| A0A3Q1ME13 | 2409676353.12 | 226034292.94 | 3.41 | 0.92 | 0.03 |
| A0A3Q1MJT2 | 9084558158.93 | 794481935.38 | 3.52 | 0.92 | 0.03 |
| A0A3Q1MK76 | 11242137855.57 | 1596195247.01 | 2.82 | 0.92 | 0.03 |
| A0A3Q1ML66 | 458170835.29 | 23015810.64 | 4.32 | 0.98 | 0.01 |
| A0A3Q1ML69 | 1295955578.20 | 26904426.21 | 5.59 | 1.00 | 0.00 |
| A0A3Q1MQA8 | 136232051.03 | 6043175.26 | 4.49 | 0.99 | 0.00 |
| A0A3Q1MRT9 | 1274045907.77 | 134670384.83 | 3.24 | 0.93 | 0.03 |
| A0A3Q1MS08 | 103491797.42 | 13650446.67 | 2.92 | 0.91 | 0.03 |
| A0A3Q1MSP7 | 34836775.02 | 672670.06 | 5.69 | 0.99 | 0.00 |
| A0A3Q1MT88 | 1965842420.82 | 268816097.71 | 2.87 | 0.93 | 0.03 |
| A0A3Q1MUH4 | 203273157.68 | 12850779.43 | 3.98 | 0.98 | 0.01 |
| A0A3Q1MUS6 | 3365564349.39 | 364889084.77 | 3.21 | 0.95 | 0.02 |
| A0A3Q1MWD4 | 17183650263.47 | 257948773.31 | 6.06 | 1.00 | 0.00 |
| A0A3Q1MYK9 | 11754055853.86 | 130119879.92 | 6.50 | 1.00 | 0.00 |
| A0A3Q1N0K9 | 2317856135.56 | 81549063.12 | 4.83 | 0.86 | 0.05 |
| A0A3Q1N363 | 1055930366.26 | 172925755.73 | 2.61 | 0.86 | 0.05 |
| A0A3Q1N4S4 | 10792331926.16 | 734570258.42 | 3.88 | 0.98 | 0.01 |
| A0A3Q1N7H1 | 7707253355.52 | 689199433.15 | 3.48 | 0.96 | 0.01 |
| A0A3Q1NE13 | 60335499.01 | 5571460.72 | 3.44 | 0.91 | 0.03 |
| A0A3Q1NK73 | 993186990.52 | 116505701.31 | 3.09 | 0.93 | 0.03 |
| A0A452DHL8 | 1550047003.73 | 136230781.57 | 3.51 | 0.95 | 0.02 |
| A0A452DHZ7 | 29564507.94 | 730389.97 | 5.34 | 0.99 | 0.00 |
| A0A452DIB6 | 938226651.68 | 93280235.92 | 3.33 | 0.94 | 0.02 |
| A0A452DIE3 | 916062330.56 | 111956020.29 | 3.03 | 0.88 | 0.04 |
| A0A452DIF2 | 2769207564.12 | 338442965.58 | 3.03 | 0.90 | 0.04 |
| A0A452DII8 | 20957064723.23 | 701076777.66 | 4.90 | 0.95 | 0.02 |
| A0A452DIN2 | 5888265701.34 | 86407316.46 | 6.09 | 1.00 | 0.00 |
| A0A452DJC8 | 12863368799.19 | 789135564.34 | 4.03 | 0.98 | 0.01 |
| A0A452DK61 | 2077230043.58 | 101158480.91 | 4.36 | 0.95 | 0.02 |
| A1A4M2 | 147436798.59 | 4816636.14 | 4.94 | 0.97 | 0.01 |
| A1XEF0 | 17442556.71 | 453362.18 | 5.27 | 0.98 | 0.01 |
| A2VDM7 | 967751411.13 | 66277170.04 | 3.87 | 0.93 | 0.03 |
| A2VE07 | 141324254.54 | 3450219756.66 | -4.61 | 0.96 | 0.02 |
| A3KN22 | 4003759202.54 | 261424884.62 | 3.94 | 0.93 | 0.03 |
| A3KN51 | 1230916468.40 | 112189443.50 | 3.46 | 0.95 | 0.02 |
| A4FUI1 | 3651702267.86 | 254448939.29 | 3.84 | 0.97 | 0.01 |
| A4FUI2 | 54386649.66 | 1964240.86 | 4.79 | 0.98 | 0.01 |
| A4FV94 | 2550486053.17 | 155610970.35 | 4.03 | 0.98 | 0.01 |
| A4IFA4 | 14106663221.02 | 1174836932.91 | 3.59 | 0.91 | 0.03 |
| A5D7K5 | 699694317.56 | 20918596.37 | 5.06 | 0.87 | 0.04 |
| A5D9H1 | 2139327519.07 | 255380027.77 | 3.07 | 0.88 | 0.04 |
| A5PJB8 | 6452529260.09 | 357801244.59 | 4.17 | 0.86 | 0.05 |
| A5PJU9 | 1716613076.55 | 86403287.24 | 4.31 | 0.97 | 0.01 |
| A6QLZ0 | 3177729912.00 | 429973410.89 | 2.89 | 0.86 | 0.05 |
| A6QNK8 | 1184561602.11 | 198893366.24 | 2.57 | 0.91 | 0.04 |
| A6QPD4 | 1220540378.04 | 97586402.29 | 3.64 | 0.88 | 0.04 |
| A6QQ09 | 934784927.51 | 112149025.21 | 3.06 | 0.90 | 0.04 |
| A6QR56 | 67182794.37 | 3668607.23 | 4.19 | 0.92 | 0.03 |
| A7YW37 | 458845431.60 | 82019738345.07 | -7.48 | 1.00 | 0.00 |
| A7YWU4 | 310611857.38 | 6597147518.53 | -4.41 | 0.99 | 0.00 |
| A7YY24 | 6317157996.30 | 499950803.02 | 3.66 | 0.97 | 0.01 |
| A8E4P2 | 1651020458.55 | 91273621.96 | 4.18 | 0.98 | 0.01 |
| A8E4R4 | 85815108.82 | 2120777.89 | 5.34 | 1.00 | 0.00 |
| B5B0D4 | 72516753632.66 | 681141375.52 | 6.73 | 0.92 | 0.03 |
| B6VAP7 | 1934492134.83 | 77515063.27 | 4.64 | 0.94 | 0.02 |
| E1B941 | 5639252002.98 | 59321862.87 | 6.57 | 1.00 | 0.00 |
| E1BCA5 | 124590528.98 | 9762828.68 | 3.67 | 0.92 | 0.03 |
| E1BG76 | 2670107950.68 | 319358555.08 | 3.06 | 0.95 | 0.02 |
| E1BGF3 | 1379761030.69 | 149905948.10 | 3.20 | 0.93 | 0.03 |
| E1BI31 | 2201439755.71 | 147038133.95 | 3.90 | 0.93 | 0.03 |
| E1BL29 | 770078737.76 | 71427625.99 | 3.43 | 0.96 | 0.02 |
| F1MB54 | 433107159.97 | 43443031.25 | 3.32 | 0.96 | 0.01 |
| F1MB60 | 970052916.36 | 61070736.46 | 3.99 | 0.97 | 0.01 |
| F1MBQ0 | 757374610.22 | 105414638.66 | 2.84 | 0.93 | 0.03 |
| F1MCG5 | 1043581055.99 | 14147421.32 | 6.20 | 1.00 | 0.00 |
| F1MDA1 | 37191707913.45 | 2052323411.44 | 4.18 | 0.92 | 0.03 |
| F1MH27 | 544228888.64 | 29048449.54 | 4.23 | 0.89 | 0.04 |
| F1MH61 | 1654868528.97 | 181101762.86 | 3.19 | 0.95 | 0.02 |
| F1MHS5 | 2463866845.08 | 257773060.69 | 3.26 | 0.94 | 0.02 |
| F1MIH4 | 1020863876.55 | 55044487.19 | 4.21 | 0.96 | 0.02 |
| F1MJH8 | 15813446542.54 | 798440959.89 | 4.31 | 1.00 | 0.00 |
| F1MJP4 | 2470183587.88 | 293026383.10 | 3.08 | 0.94 | 0.02 |
| F1MLQ3 | 482774192.72 | 6308167.85 | 6.26 | 1.00 | 0.00 |
| F1MLW2 | 180893145.30 | 4366094931.79 | -4.59 | 0.91 | 0.04 |
| F1MLX0 | 407344321.25 | 44787426.45 | 3.19 | 0.91 | 0.04 |
| F1MM13 | 2073833270.45 | 181900224.12 | 3.51 | 0.90 | 0.04 |
| F1MM83 | 1051114175.08 | 35391614.65 | 4.89 | 0.98 | 0.01 |
| F1MQW0 | 161039363.32 | 19087929.55 | 3.08 | 0.89 | 0.04 |
| F1MRF8 | 532421249.67 | 30635214.84 | 4.12 | 0.95 | 0.02 |
| F1MSD6 | 265547673.37 | 16150846.73 | 4.04 | 0.98 | 0.01 |
| F1MSV8 | 1182739963.12 | 26822361.79 | 5.46 | 1.00 | 0.00 |
| F1MU30 | 10162896070.13 | 252889694.57 | 5.33 | 0.98 | 0.01 |
| F1MVM4 | 2069832183.11 | 53061949.97 | 5.29 | 0.94 | 0.02 |
| F1MYR5 | 2858341693.69 | 240724142.53 | 3.57 | 0.90 | 0.04 |
| F1MYS2 | 132093104.45 | 15209388.30 | 3.12 | 0.91 | 0.03 |
| F1N0H3 | 173353684.05 | 2716770.80 | 6.00 | 0.99 | 0.00 |
| F1N1C3 | 52743682.38 | 2043161.50 | 4.69 | 0.98 | 0.01 |
| F1N207 | 38649343087.19 | 279692807.06 | 7.11 | 1.00 | 0.00 |
| F1N2Q7 | 8721889795.69 | 952884469.34 | 3.19 | 0.96 | 0.02 |
| F1N373 | 303300983.55 | 18790203.19 | 4.01 | 0.98 | 0.01 |
| F1N382 | 3258617102.26 | 200773694.90 | 4.02 | 0.95 | 0.02 |
| F1N3L2 | 376252472.18 | 52587112.57 | 2.84 | 0.91 | 0.03 |
| F1N428 | 1053971155.58 | 43041340.67 | 4.61 | 0.99 | 0.00 |
| F1N431 | 16130045636.64 | 1365206773.80 | 3.56 | 0.98 | 0.01 |
| F1N4Q2 | 18318808113.48 | 2106656028.42 | 3.12 | 0.90 | 0.04 |
| F1N4R4 | 145487137.26 | 4433524.57 | 5.04 | 0.87 | 0.05 |
| F1N555 | 13064719607.74 | 415940836.10 | 4.97 | 0.94 | 0.02 |
| F1N5A4 | 570730475.53 | 36677182.20 | 3.96 | 0.97 | 0.01 |
| G3MXH9 | 2411409848.25 | 42211423.34 | 5.84 | 0.98 | 0.01 |
| G3MXZ0 | 2843718119.33 | 374991258.42 | 2.92 | 0.86 | 0.05 |
| G3N0V0 | 4805522038.57 | 812530501.48 | 2.56 | 0.86 | 0.05 |
| G3X6N3 | 2248825934.47 | 49525449.06 | 5.50 | 1.00 | 0.00 |
| G3X7D8 | 400807969.37 | 54384882.97 | 2.88 | 0.92 | 0.03 |
| G5E597 | 13888269701.19 | 831384875.75 | 4.06 | 0.98 | 0.01 |
| G5E5G3 | 10827577008.59 | 154434144.25 | 6.13 | 1.00 | 0.00 |
| K7QF88 | 1878173546.59 | 190306473.77 | 3.30 | 0.94 | 0.02 |
| O02741 | 10430969549.09 | 273777066.31 | 5.25 | 1.00 | 0.00 |
| O18778 | 1477563032.12 | 130360315.65 | 3.50 | 0.96 | 0.01 |
| O62830 | 382636653.20 | 35308002.69 | 3.44 | 0.95 | 0.02 |
| O97680 | 6351259218.60 | 412359341.29 | 3.95 | 0.94 | 0.02 |
| P00514 | 353912882.56 | 6177834.29 | 5.84 | 0.88 | 0.04 |
| P02584 | 6857746397.68 | 183324624.81 | 5.23 | 0.98 | 0.01 |
| P08165 | 470748330.50 | 57754578.11 | 3.03 | 0.89 | 0.04 |
| P10096 | 12063840665.54 | 702581068.26 | 4.10 | 0.98 | 0.01 |
| P15497 | 15507599608.81 | 1419968800.11 | 3.45 | 0.98 | 0.01 |
| P17453 | 4011622895.28 | 288616543.34 | 3.80 | 0.97 | 0.01 |
| P22226 | 14251268392.55 | 1185981121.63 | 3.59 | 0.93 | 0.02 |
| P52556 | 771189379.80 | 50101075.50 | 3.94 | 0.98 | 0.01 |
| P60712 | 88771554138.41 | 1753828461.98 | 5.66 | 1.00 | 0.00 |
| P61356 | 5667149355.18 | 354433470.63 | 4.00 | 0.98 | 0.01 |
| P68138 | 24779500052.59 | 461748344.06 | 5.75 | 1.00 | 0.00 |
| P79102 | 1242749680.30 | 75505127.98 | 4.04 | 0.99 | 0.00 |
| P79103 | 345499316.03 | 1798749.84 | 7.59 | 1.00 | 0.00 |
| P81948 | 4293152532.58 | 283513291.21 | 3.92 | 0.95 | 0.02 |
| Q02373 | 1250959332.55 | 170329614.38 | 2.88 | 0.96 | 0.01 |
| Q08D83 | 132107950.38 | 10876931.27 | 3.60 | 0.96 | 0.01 |
| Q08D98 | 25572113519.23 | 2882774594.39 | 3.15 | 0.95 | 0.02 |
| Q08DU9 | 1412736536.13 | 121249224.03 | 3.54 | 0.98 | 0.01 |
| Q08E11 | 345358433.66 | 12475897.67 | 4.79 | 0.93 | 0.02 |
| Q0IIG8 | 2011757141.36 | 189332003.65 | 3.41 | 0.97 | 0.01 |
| Q0P5D6 | 1572960592.28 | 133235683.34 | 3.56 | 0.96 | 0.02 |
| Q0PHW6 | 1281803208.37 | 90689181.40 | 3.82 | 0.99 | 0.00 |
| Q0VC02 | 648507921.29 | 84118557.22 | 2.95 | 0.87 | 0.05 |
| Q0VD50 | 3124686061.07 | 183138887.81 | 4.09 | 0.99 | 0.00 |
| Q148C8 | 3790947888.49 | 187928993.28 | 4.33 | 0.93 | 0.03 |
| Q148D6 | 319959744.43 | 22339781.82 | 3.84 | 0.99 | 0.00 |
| Q148J4 | 203585619.71 | 15869020.76 | 3.68 | 0.95 | 0.02 |
| Q17QL8 | 546644378.29 | 72549212.47 | 2.91 | 0.89 | 0.04 |
| Q1LZ90 | 451434523.98 | 15624922.31 | 4.85 | 0.99 | 0.01 |
| Q1LZD8 | 706192234.96 | 21279694.28 | 5.05 | 0.98 | 0.01 |
| Q29443 | 1449790668.72 | 70557352.40 | 4.36 | 1.00 | 0.00 |
| Q2HJ88 | 325964712.71 | 27265756.09 | 3.58 | 0.97 | 0.01 |
| Q2KHU0 | 408080788.80 | 12832566.01 | 4.99 | 0.99 | 0.00 |
| Q2KI62 | 6853498419.81 | 270973121.96 | 4.66 | 1.00 | 0.00 |
| Q2KIQ7 | 287980962.32 | 19050442.70 | 3.92 | 0.98 | 0.01 |
| Q2KJG4 | 405069914.89 | 18452821.39 | 4.46 | 0.99 | 0.01 |
| Q2NL04 | 2923382526.70 | 117021616.79 | 4.64 | 0.99 | 0.00 |
| Q2NL34 | 1698517850.89 | 126616781.26 | 3.75 | 0.97 | 0.01 |
| Q2UVX4 | 55359656495.15 | 5391593845.85 | 3.36 | 0.97 | 0.01 |
| Q2YDE4 | 1843942688.57 | 251001383.69 | 2.88 | 0.89 | 0.04 |
| Q2YDG3 | 672874435.64 | 73027547.29 | 3.20 | 0.96 | 0.02 |
| Q2YDH6 | 1392042636.19 | 105122973.59 | 3.73 | 0.98 | 0.01 |
| Q32KW2 | 3515358765.50 | 72026337.74 | 5.61 | 0.99 | 0.00 |
| Q32L41 | 1652433569.29 | 116343258.46 | 3.83 | 0.93 | 0.03 |
| Q32LG5 | 869878486.63 | 38032577.36 | 4.52 | 1.00 | 0.00 |
| Q32PB9 | 79360709.85 | 10753413.57 | 2.88 | 0.87 | 0.05 |
| Q32PG0 | 6534697236.36 | 431269652.23 | 3.92 | 0.98 | 0.01 |
| Q3SWY2 | 1587776226.06 | 95929859.76 | 4.05 | 0.92 | 0.03 |
| Q3SZ62 | 6423004860.59 | 933974591.48 | 2.78 | 0.89 | 0.04 |
| Q3SZP7 | 44173338315.49 | 5049858003.97 | 3.13 | 0.88 | 0.04 |
| Q3ZBD9 | 2558190626.76 | 303042007.45 | 3.08 | 0.89 | 0.04 |
| Q3ZBF2 | 565535394.32 | 68232581.40 | 3.05 | 0.87 | 0.04 |
| Q3ZBF3 | 2593358282.23 | 68840761.99 | 5.24 | 1.00 | 0.00 |
| Q3ZBH8 | 3124230471.61 | 378674811.73 | 3.04 | 0.92 | 0.03 |
| Q3ZBN8 | 820576258.31 | 37387355.28 | 4.46 | 1.00 | 0.00 |
| Q3ZBY8 | 2819608036.49 | 272288410.62 | 3.37 | 0.98 | 0.01 |
| Q3ZC02 | 3034681190.58 | 199236640.17 | 3.93 | 0.89 | 0.04 |
| Q3ZCD3 | 161095564.41 | 6565615.43 | 4.62 | 1.00 | 0.00 |
| Q3ZCG9 | 1359961457.38 | 137706481.01 | 3.30 | 0.95 | 0.02 |
| Q3ZCJ9 | 9261091273.16 | 459485271.75 | 4.33 | 0.99 | 0.00 |
| Q45LU9 | 497193860.81 | 13954660.45 | 5.15 | 0.93 | 0.03 |
| Q56JX9 | 14463357519.09 | 420718091.74 | 5.10 | 0.93 | 0.03 |
| Q58CT1 | 454713644.18 | 16790253.37 | 4.76 | 0.96 | 0.02 |
| Q58DT1 | 2483115910.20 | 286564173.43 | 3.12 | 0.92 | 0.03 |
| Q5BIR5 | 1851820977.18 | 147026178.03 | 3.65 | 0.92 | 0.03 |
| Q5E971 | 983204555.77 | 56350389.25 | 4.12 | 0.99 | 0.00 |
| Q5E9C2 | 196723483.02 | 14039175.24 | 3.81 | 0.93 | 0.02 |
| Q5E9E8 | 29159325.14 | 353569.44 | 6.37 | 1.00 | 0.00 |
| Q5E9T4 | 111959592.56 | 5378962.80 | 4.38 | 0.96 | 0.01 |
| Q6EWQ7 | 4646711915.97 | 297662102.29 | 3.96 | 0.96 | 0.02 |
| Q6H320 | 79413465.15 | 1141981002.07 | -3.85 | 0.91 | 0.03 |
| Q6JTG5 | 655444577.94 | 6266829.70 | 6.71 | 1.00 | 0.00 |
| Q862I1 | 6026994801.21 | 144492315.47 | 5.38 | 0.88 | 0.04 |
| Q8SNJ1 | 358647224.60 | 1580625.01 | 7.83 | 0.90 | 0.04 |
| Q95140 | 239000849.73 | 2061374.91 | 6.86 | 0.95 | 0.02 |
| Q95KU9 | 412174484.80 | 28803186.86 | 3.84 | 0.87 | 0.05 |
| Q9TR53 | 43014316.71 | 1467232.19 | 4.87 | 0.98 | 0.01 |
| Q9TRQ0 | 200115691.34 | 8697018.42 | 4.52 | 0.94 | 0.02 |
| Q9TU25 | 132083502.52 | 7398130.01 | 4.16 | 0.97 | 0.01 |
| Q9XSC9 | 997116714.82 | 111319366.32 | 3.16 | 0.95 | 0.02 |

^1^Treatments: 1)C ( Control group) treatment: fed a basal diet and not challenged with *E. coli* K99; 2) D (positive control, Diarrhea group): fed a basal diet and orally challenged with *E. coli* K99 (30 mL; 1×10^9^ CFU/mL) and Antibiotic support therapy（Intramuscular gentamicin 20 mL/ d）;

Supplemental Table S8 Gene ontology enrichment analysis shows the host proteins demonstrated that the mostly enriched biological functions

| GOTerm | ID | Ontology | Count | Qvalue | Groups |
| --- | --- | --- | --- | --- | --- |
| extracellular region | GO:0005576 | CC | 42 | 1.93734E-18 | H |
| extracellular space | GO:0005615 | CC | 36 | 2.76212E-16 | H |
| extracellular exosome | GO:0070062 | CC | 32 | 2.76212E-16 | H |
| extracellular vesicle | GO:1903561 | CC | 32 | 2.76212E-16 | H |
| extracellular organelle | GO:0043230 | CC | 32 | 2.76212E-16 | H |
| cytoplasm | GO:0005737 | CC | 55 | 1.08302E-13 | H |
| vesicle | GO:0031982 | CC | 34 | 1.08302E-13 | H |
| intracellular | GO:0005622 | CC | 59 | 3.62443E-10 | H |
| cellular process | GO:0009987 | BP | 61 | 2.97773E-09 | H |
| catalytic activity | GO:0003824 | MF | 41 | 9.78453E-09 | H |
| membrane-bounded organelle | GO:0043227 | CC | 51 | 2.16617E-08 | H |
| organelle | GO:0043226 | CC | 53 | 3.27653E-08 | H |
| metabolic process | GO:0008152 | BP | 49 | 7.29619E-08 | H |
| cellular metabolic process | GO:0044237 | BP | 46 | 8.88405E-08 | H |
| small molecule binding | GO:0036094 | MF | 23 | 4.41765E-07 | H |
| organonitrogen compound metabolic process | GO:1901564 | BP | 34 | 4.87574E-07 | H |
| organic substance metabolic process | GO:0071704 | BP | 45 | 4.92099E-07 | H |
| oxidation-reduction process | GO:0055114 | BP | 15 | 1.81113E-06 | H |
| proteasome core complex | GO:0005839 | CC | 5 | 1.81113E-06 | H |
| threonine-type endopeptidase activity | GO:0004298 | MF | 5 | 2.05248E-06 | H |
| threonine-type peptidase activity | GO:0070003 | MF | 5 | 2.05248E-06 | H |
| mitochondrion | GO:0005739 | CC | 18 | 2.36726E-06 | H |
| oxidoreductase activity | GO:0016491 | MF | 13 | 5.58719E-06 | H |
| intracellular organelle | GO:0043229 | CC | 45 | 6.15195E-06 | H |
| small molecule metabolic process | GO:0044281 | BP | 16 | 6.15195E-06 | H |
| myelin sheath | GO:0043209 | CC | 7 | 7.92133E-06 | H |
| primary metabolic process | GO:0044238 | BP | 41 | 1.05591E-05 | H |
| catabolic process | GO:0009056 | BP | 17 | 1.34335E-05 | H |
| cellular amide metabolic process | GO:0043603 | BP | 13 | 1.34335E-05 | H |
| binding | GO:0005488 | MF | 45 | 1.38688E-05 | H |
| extracellular region | GO:0005576 | CC | 44 | 2.10412E-20 | D |
| extracellular space | GO:0005615 | CC | 39 | 6.34296E-19 | D |
| extracellular exosome | GO:0070062 | CC | 34 | 2.50338E-18 | D |
| extracellular vesicle | GO:1903561 | CC | 34 | 2.50338E-18 | D |
| extracellular organelle | GO:0043230 | CC | 34 | 2.50338E-18 | D |
| cytoplasm | GO:0005737 | CC | 51 | 6.13343E-11 | D |
| vesicle | GO:0031982 | CC | 37 | 2.48194E-16 | D |
| intracellular | GO:0005622 | CC | 57 | 6.52308E-09 | D |
| cellular process | GO:0009987 | BP | 58 | 1.23579E-07 | D |
| catalytic activity | GO:0003824 | MF | 37 | 1.77723E-06 | D |
| membrane-bounded organelle | GO:0043227 | CC | 52 | 6.87103E-09 | D |
| organelle | GO:0043226 | CC | 54 | 1.00442E-08 | D |
| metabolic process | GO:0008152 | BP | 40 | 0.000929228 | D |
| cellular metabolic process | GO:0044237 | BP | 37 | 0.00114128 | D |
| organic substance metabolic process | GO:0071704 | BP | 38 | 0.000811915 | D |
| mitochondrion | GO:0005739 | CC | 16 | 8.49818E-05 | D |
| intracellular organelle | GO:0043229 | CC | 46 | 3.0749E-06 | D |
| small molecule metabolic process | GO:0044281 | BP | 13 | 0.00096989 | D |
| myelin sheath | GO:0043209 | CC | 8 | 7.07933E-07 | D |
| cellular amide metabolic process | GO:0043603 | BP | 11 | 0.000750681 | D |
| binding | GO:0005488 | MF | 47 | 2.90151E-06 | D |
| extracellular region | GO:0005576 | CC | 39 | 1.20971E-15 | P |
| extracellular space | GO:0005615 | CC | 31 | 1.49385E-11 | P |
| extracellular exosome | GO:0070062 | CC | 27 | 1.49385E-11 | P |
| extracellular vesicle | GO:1903561 | CC | 27 | 1.49385E-11 | P |
| extracellular organelle | GO:0043230 | CC | 27 | 1.49385E-11 | P |
| cytoplasm | GO:0005737 | CC | 49 | 1.43641E-09 | P |
| vesicle | GO:0031982 | CC | 28 | 8.11607E-09 | P |
| intracellular | GO:0005622 | CC | 55 | 9.25762E-08 | P |
| cellular process | GO:0009987 | BP | 57 | 5.15043E-07 | P |
| membrane-bounded organelle | GO:0043227 | CC | 45 | 1.7147E-05 | P |
| organelle | GO:0043226 | CC | 50 | 1.11136E-06 | P |
| cellular metabolic process | GO:0044237 | BP | 41 | 2.32934E-05 | P |
| organonitrogen compound metabolic process | GO:1901564 | BP | 31 | 1.83099E-05 | P |
| mitochondrion | GO:0005739 | CC | 17 | 1.53543E-05 | P |
| cellular amide metabolic process | GO:0043603 | BP | 15 | 5.85984E-07 | P |
| amide biosynthetic process | GO:0043604 | BP | 13 | 2.43378E-06 | P |
| translation | GO:0006412 | BP | 13 | 5.85984E-07 | P |
| peptide metabolic process | GO:0006518 | BP | 14 | 4.42453E-07 | P |
| organonitrogen compound biosynthetic process | GO:1901566 | BP | 18 | 6.09974E-07 | P |
| peptide biosynthetic process | GO:0043043 | BP | 13 | 6.41854E-07 | P |
| intracellular non-membrane-bounded organelle | GO:0043232 | CC | 24 | 1.28973E-05 | P |
| non-membrane-bounded organelle | GO:0043228 | CC | 24 | 1.28973E-05 | P |
| defense response to bacterium | GO:0042742 | BP | 7 | 1.54647E-05 | P |
| response to stress | GO:0006950 | BP | 20 | 2.78635E-05 | P |
| response to other organism | GO:0051707 | BP | 12 | 2.78791E-05 | P |
| defense response to other organism | GO:0098542 | BP | 11 | 2.78791E-05 | P |
| structural constituent of ribosome | GO:0003735 | MF | 8 | 2.78791E-05 | P |
| response to external biotic stimulus | GO:0043207 | BP | 12 | 2.78791E-05 | P |
| mitochondrial inner membrane | GO:0005743 | CC | 10 | 2.78791E-05 | P |
| response to biotic stimulus | GO:0009607 | BP | 12 | 3.48138E-05 | P |

Supplemental Table S9 Neonatal calves proteins and microbial functions that were differentially abundant in calf infection with *E.coli* K99 used by Spearman's correlation

| Microbial protein | Host protein |  |  |
| --- | --- | --- | --- |
| node1 | node2 | Spearman r | pvalue |
| A0A3Q1LSI9 | ENOG410XQ0R | 1 | 0 |
| A0A3Q1MYK9 | ENOG410XQ0R | 1 | 0 |
| A6QM03 | ENOG410XQ0R | 1 | 0 |
| Q56JX9 | ENOG410XQ0R | 1 | 0 |
| COG5059 | COG5059 | 1 | 0 |
| COG0017 | COG5059 | -1 | 0 |
| E1BCE0 | COG5059 | 1 | 0 |
| F1N0H3 | COG5059 | -1 | 0 |
| P62248 | COG5059 | -1 | 0 |
| Q3ZC02 | COG5059 | -1 | 0 |
| Q9XSC9 | COG5059 | -1 | 0 |
| A0A3Q1LSI9 | COG4573 | 1 | 0 |
| A0A3Q1MYK9 | COG4573 | 1 | 0 |
| A6QM03 | COG4573 | 1 | 0 |
| Q56JX9 | COG4573 | 1 | 0 |
| A0A3Q1LSI9 | COG1012..COG1454 | 1 | 0 |
| A0A3Q1MYK9 | COG1012..COG1454 | 1 | 0 |
| A6QM03 | COG1012..COG1454 | 1 | 0 |
| Q56JX9 | COG1012..COG1454 | 1 | 0 |
| COG5059 | COG0443 | -1 | 0 |
| COG0017 | COG0443 | 1 | 0 |
| E1BCE0 | COG0443 | -1 | 0 |
| F1N0H3 | COG0443 | 1 | 0 |
| P62248 | COG0443 | 1 | 0 |
| Q3ZC02 | COG0443 | 1 | 0 |
| Q9XSC9 | COG0443 | 1 | 0 |
| COG5059 | COG0017 | -1 | 0 |
| COG0017 | COG0017 | 1 | 0 |
| E1BCE0 | COG0017 | -1 | 0 |
| F1N0H3 | COG0017 | 1 | 0 |
| P62248 | COG0017 | 1 | 0 |
| Q3ZC02 | COG0017 | 1 | 0 |
| Q9XSC9 | COG0017 | 1 | 0 |
| A0A3Q1LSI9 | A0A3Q1LSI9 | 1 | 0 |
| A0A3Q1MYK9 | A0A3Q1LSI9 | 1 | 0 |
| A6QM03 | A0A3Q1LSI9 | 1 | 0 |
| Q56JX9 | A0A3Q1LSI9 | 1 | 0 |
| A0A3Q1LSI9 | A0A3Q1MYK9 | 1 | 0 |
| A0A3Q1MYK9 | A0A3Q1MYK9 | 1 | 0 |
| A6QM03 | A0A3Q1MYK9 | 1 | 0 |
| Q56JX9 | A0A3Q1MYK9 | 1 | 0 |
| A0A3Q1LSI9 | A6QM03 | 1 | 0 |
| A0A3Q1MYK9 | A6QM03 | 1 | 0 |
| A6QM03 | A6QM03 | 1 | 0 |
| Q56JX9 | A6QM03 | 1 | 0 |
| COG5059 | E1BCE0 | 1 | 0 |
| COG0017 | E1BCE0 | -1 | 0 |
| E1BCE0 | E1BCE0 | 1 | 0 |
| F1N0H3 | E1BCE0 | -1 | 0 |
| P62248 | E1BCE0 | -1 | 0 |
| Q3ZC02 | E1BCE0 | -1 | 0 |
| Q9XSC9 | E1BCE0 | -1 | 0 |
| A0A3Q1LSI9 | F1MVM4 | 1 | 0 |
| A0A3Q1MYK9 | F1MVM4 | 1 | 0 |
| A6QM03 | F1MVM4 | 1 | 0 |
| Q56JX9 | F1MVM4 | 1 | 0 |
| COG5059 | F1N0H3 | -1 | 0 |
| COG0017 | F1N0H3 | 1 | 0 |
| E1BCE0 | F1N0H3 | -1 | 0 |
| F1N0H3 | F1N0H3 | 1 | 0 |
| P62248 | F1N0H3 | 1 | 0 |
| Q3ZC02 | F1N0H3 | 1 | 0 |
| Q9XSC9 | F1N0H3 | 1 | 0 |
| COG5059 | P62248 | -1 | 0 |
| COG0017 | P62248 | 1 | 0 |
| E1BCE0 | P62248 | -1 | 0 |
| F1N0H3 | P62248 | 1 | 0 |
| P62248 | P62248 | 1 | 0 |
| Q3ZC02 | P62248 | 1 | 0 |
| Q9XSC9 | P62248 | 1 | 0 |
| COG5059 | Q3ZBF3 | -1 | 0 |
| COG0017 | Q3ZBF3 | 1 | 0 |
| E1BCE0 | Q3ZBF3 | -1 | 0 |
| F1N0H3 | Q3ZBF3 | 1 | 0 |
| P62248 | Q3ZBF3 | 1 | 0 |
| Q3ZC02 | Q3ZBF3 | 1 | 0 |
| Q9XSC9 | Q3ZBF3 | 1 | 0 |
| COG5059 | Q3ZC02 | -1 | 0 |
| COG0017 | Q3ZC02 | 1 | 0 |
| E1BCE0 | Q3ZC02 | -1 | 0 |
| F1N0H3 | Q3ZC02 | 1 | 0 |
| P62248 | Q3ZC02 | 1 | 0 |
| Q3ZC02 | Q3ZC02 | 1 | 0 |
| Q9XSC9 | Q3ZC02 | 1 | 0 |
| A0A3Q1LSI9 | Q56JX9 | 1 | 0 |
| A0A3Q1MYK9 | Q56JX9 | 1 | 0 |
| A6QM03 | Q56JX9 | 1 | 0 |
| Q56JX9 | Q56JX9 | 1 | 0 |
| COG5059 | Q9XSC9 | -1 | 0 |
| COG0017 | Q9XSC9 | 1 | 0 |
| E1BCE0 | Q9XSC9 | -1 | 0 |
| F1N0H3 | Q9XSC9 | 1 | 0 |
| P62248 | Q9XSC9 | 1 | 0 |
| Q3ZC02 | Q9XSC9 | 1 | 0 |
| Q9XSC9 | Q9XSC9 | 1 | 0 |





Supplemental Figure1-1 Comparsion of digest-associate bacterial profiles with Principal Coordinate (PCoA). PCoA plot was generated using Bray_Curtis for bacterial and fungal species in different intestinal contents samples of neonatal calves (bacterial(A, Duodenum；B, Jejunum; C, Ileum; D, Cecum; E, Colon; F, Rectum). (CD, Control Duodenum; DD, Diarrhea Duodenum; PD, MSP Duodenum; CJ, Control Jejunum; DJ, Diarrhea Jejunum; PJ, MSP Jejunum; CI, Control Ileum; DI, Diarrhea Ileum; PI, MSP Ileum; CCa, Control Cecum; DCa, Diarrhea Cecum; PCa, MSP Cecum; CCo, Control Colon; DCo, Diarrhea Colon; PCo, MSP Colon; CRe, Control Rectum; DRe, Diarrhea Rectum; PRe, MSP Rectum)


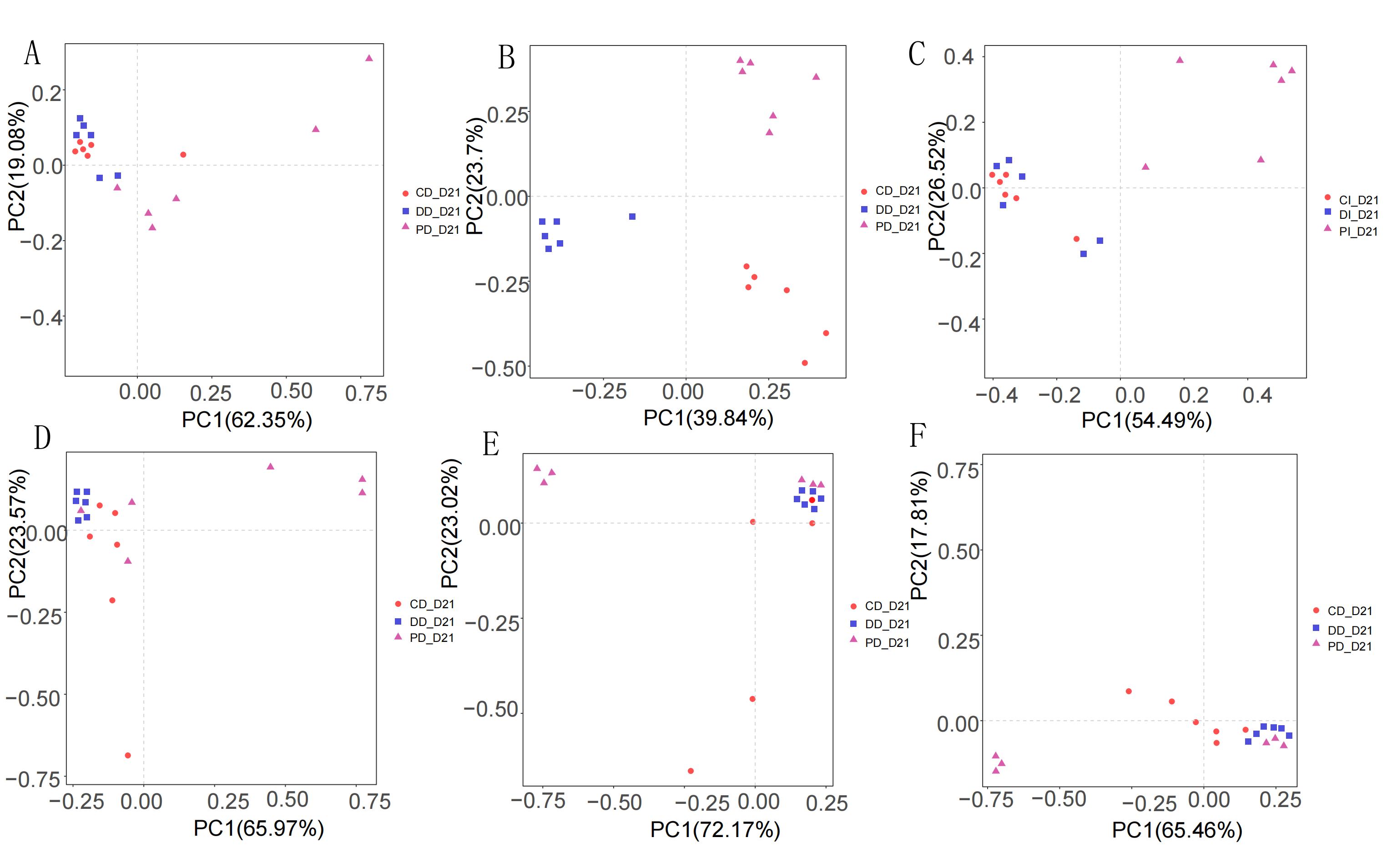


Supplemental Figure1-2 Comparsion of digest-associate bacterial profiles with Principal Coordinate (PCoA). PCoA plot was generated using Bray_Curtis for bacterial and fungal species in different intestinal contents samples of neonatal calves (fungi(A, Duodenum；B, Jejunum; C, Ileum; D, Cecum; E, Colon; F, Rectum). (CD, Control Duodenum; DD, Diarrhea Duodenum; PD, MSP Duodenum; CJ, Control Jejunum; DJ, Diarrhea Jejunum; PJ, MSP Jejunum; CI, Control Ileum; DI, Diarrhea Ileum; PI, MSP Ileum; CCa, Control Cecum; DCa, Diarrhea Cecum; PCa, MSP Cecum; CCo, Control Colon; DCo, Diarrhea Colon; PCo, MSP Colon; CRe, Control Rectum; DRe, Diarrhea Rectum; PRe, MSP Rectum


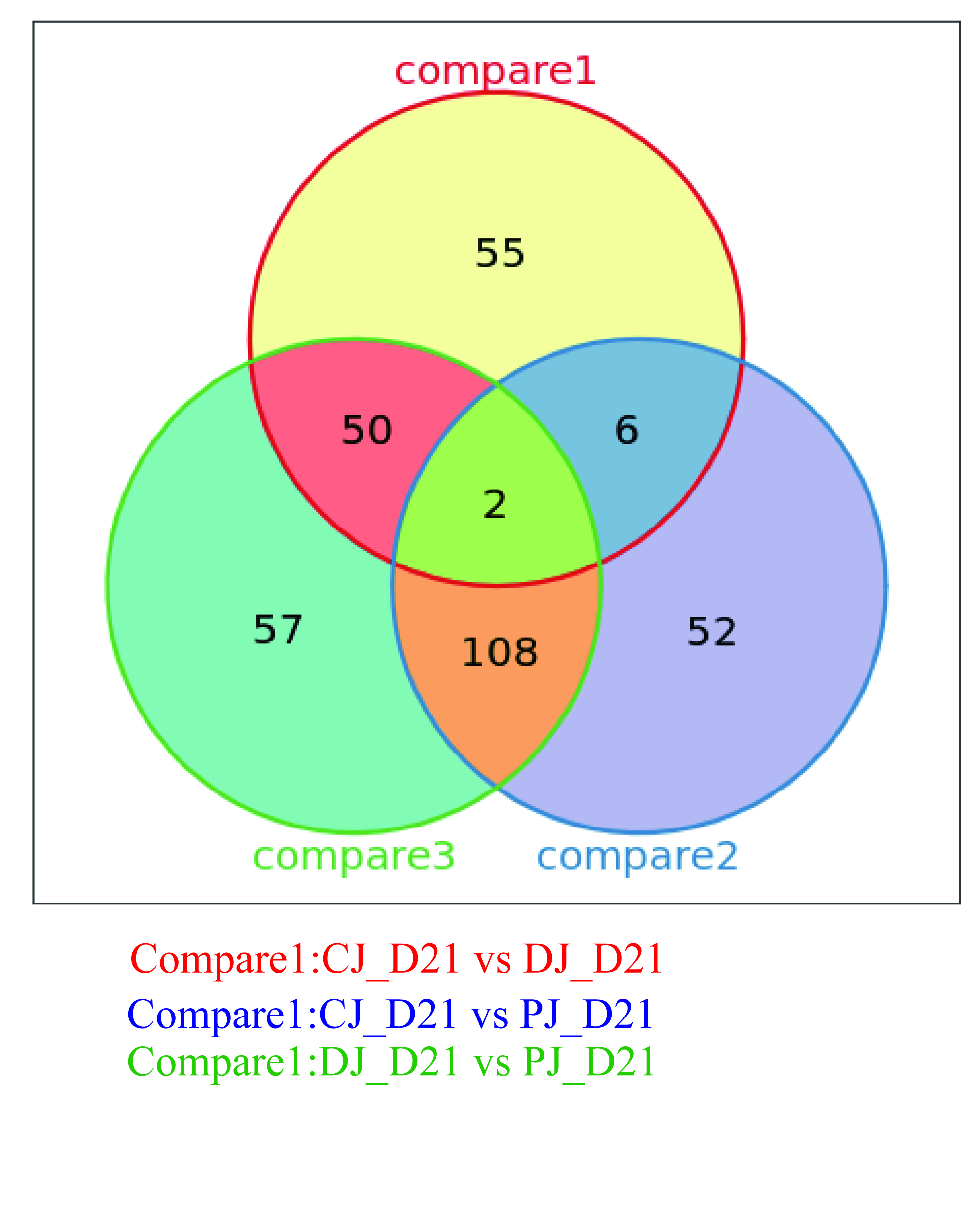


Supplemental Figure 2 Venn diagram shows the host differential proteins among control, D and P groups.


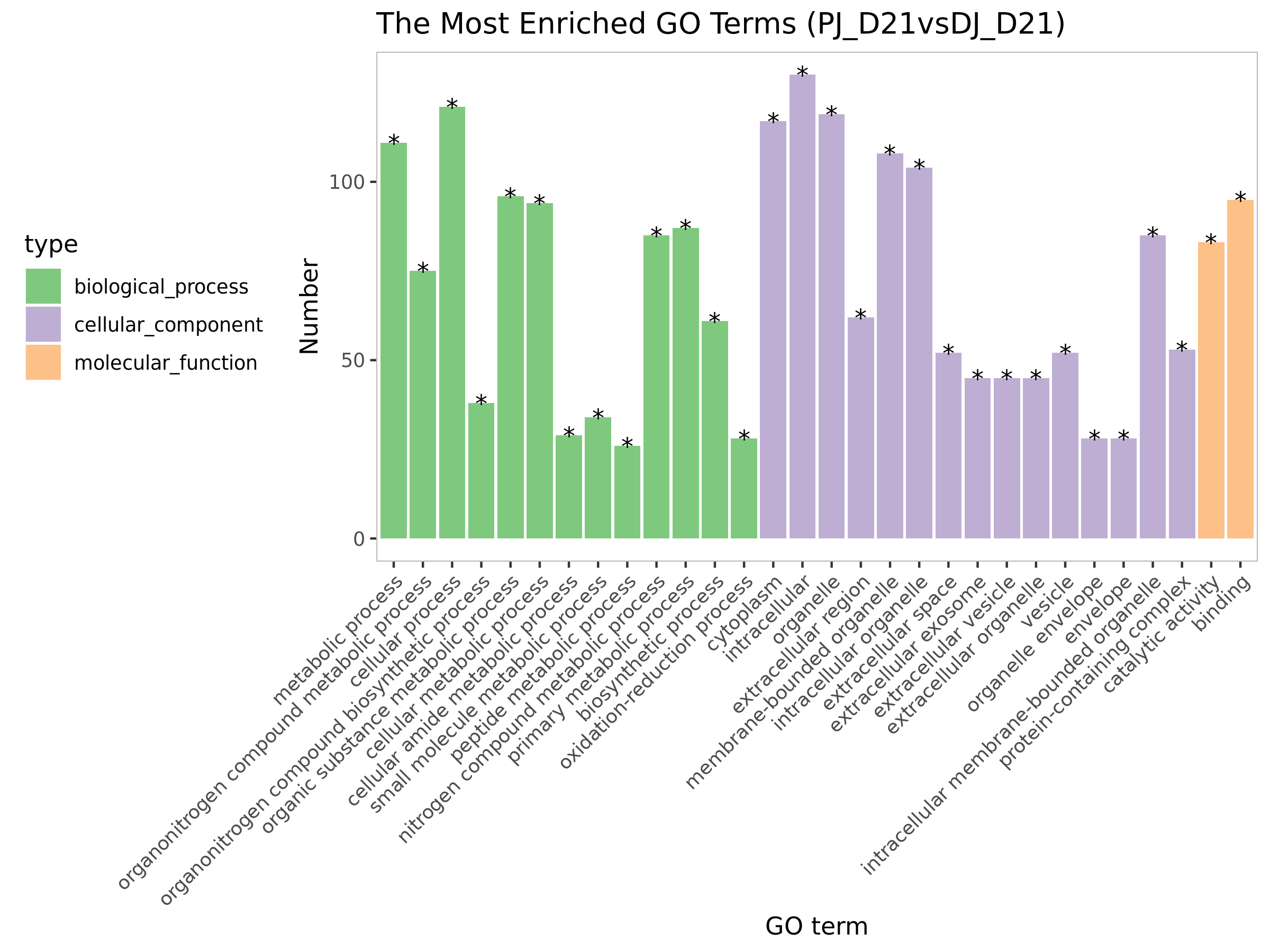


Supplemental Figure 3 Gene ontology enrichment analysis shows the host proteins demonstrated that the mostly enriched biological functions


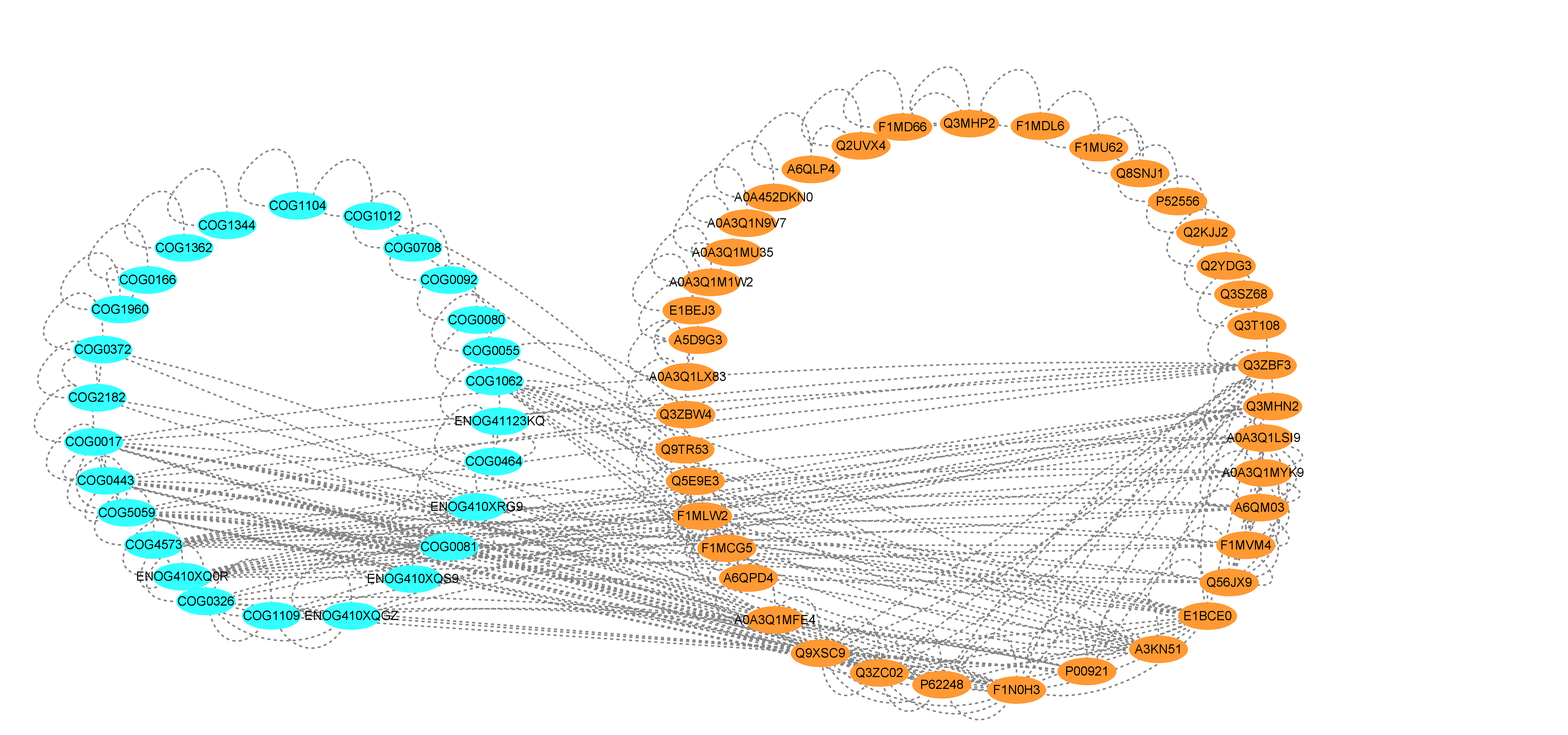


Supplemental Figure 4 Network of co-occurrence of neonatal calves proteins and microbial functions that were differentially abundant in calf infection with *E.coli* K99. Spearman's correlation was used for constructing the network and the edge indicates a Spearman's pvalue of > 0.7 or < -0.7 and p < 0.05. The size of nodes is proportional to the number of connections and the thickness of edges indicates the Spearman’s r values. Blue color indicates microbial COG while yellow indicates neonatal calves.
